# Posterior parietal cortex mediates rarity-induced decision bias

**DOI:** 10.1093/nsr/nwag449

**Published:** 2026-07-21

**Authors:** Weihao Sheng, Xinrui Huang, Yang Xie, Manolis C Tsakiris, Yang Yang

**Affiliations:** School of Life Science and Technology & State Key Laboratory of Advanced Medical Materials and Devices, ShanghaiTech University, Shanghai 201210, China; School of Life Science and Technology & State Key Laboratory of Advanced Medical Materials and Devices, ShanghaiTech University, Shanghai 201210, China; Global Institute of Future Technology, Shanghai Jiao Tong University, Shanghai 200240, China; Academy of Mathematics and Systems Science, Chinese Academy of Sciences, Beijing 100190, China; School of Life Science and Technology & State Key Laboratory of Advanced Medical Materials and Devices, ShanghaiTech University, Shanghai 201210, China

**Keywords:** rarity, probabilistic decision-making, uncertainty, bias, posterior parietal cortex

## Abstract

Optimal decision-making under uncertainty is critical for survival, yet real-world decisions often deviate from optimality. Here, we report a rarity-induced bias in humans and mice, where rare events exert a stronger and more persistent impact on subsequent decisions than common events. Optogenetic manipulations reveal distinct learning stage-specific roles of the posterior parietal cortex (PPC): in the naïve stage, PPC drives the rarity-induced bias and interferes with optimal decisions; in the expert stage, PPC becomes essential for stimulus-based decisions. *In vivo* recordings support these dynamic roles, showing that rare reward history bidirectionally modulates choice-selective PPC neurons to induce bias, while learning enhances stimulus-encoding of PPC neurons to promote optimal decisions. A data-inspired dual-agent model recapitulated the bias and learning process, and predicted PPC’s contribution to learning as confirmed by optogenetics. Our study uncovers a rarity-induced bias, and reveals PPC’s essential roles in mediating this bias and stimulus-based learning under uncertainty.

## INTRODUCTION

In a world that is probabilistic in nature, the ability to learn from uncertain outcomes and make optimal decisions is essential for animals’ survival and well-being [1–3]. Bayesian models provide a normative framework for optimal behaviors in uncertain environments [4]. However, decisions are not always optimal: psychological and economic studies have reported systematic deviations from optimal behavior, one important example being decisions involving rare probabilities [5,6]. Specifically, people tend to overweight rare probabilities, acting as if rare events are more likely to occur than they really are [5,6]. This phenomenon of rarity overweighting has significant real-life implications, fundamental to economic behaviors such as gambling and insurance purchasing [7,8]. Psychologists and economists have attributed this behavioral bias to various factors, including the recency, representativeness, novelty, and saliency of rare events [5,9–11], as well as the emotional, psychological, and cognitive states of decision-makers [12–14].

On top of these factors, a defining feature of all rare events is simply the probabilistic rarity itself: rare events occur less frequently than common events. Surprisingly, this inherent quantitative property has been largely overlooked, possibly because it is inevitably entangled with other factors in behavioral experiments designed under the *expected utility* or *prospect theory* framework [6,15]. In these paradigms, rarity is often complicated with significant risk, which on its own can influence decisions [13,16,17], and rare outcomes are typically paired with larger magnitudes of gain or loss [1,3,10,18], causing rarity to be confounded with saliency. As a result, whether the probabilistic rarity, isolated from other factors, has any impact on decision-making remains unknown, and the underlying neural mechanisms have not been explored.

**Table 1.** Glossary.

**Term**	**Abbrev.**
Posterior parietal cortex	PPC
Choice repeat probability for the next same-stimulus trial	sRP***_n+k+1_***
Choice repeat probability for the next trial	nRP***_n+1_***
Optogenetic inhibition during stimulus and choice epoch of rare trials	*stim*&*choice-opto_rare_*
Optogenetic inhibition during outcome epoch of rare trials	*outcome-opto_rare_*
Optogenetic inhibition during stimulus and choice epoch of common trials	*stim*&*choice-opto_comm_*
Rare reward Modulation Index	RMI
Choice selectivity index	CSI
Stimulus selectivity index	SSI

To process the quantitative information of rarity and use it to drive decisions, the relevant brain regions and neural circuits should be able to: (1) determine the frequency of event occurrence by assessing the probability from event history; (2) use this quantitative information to guide future decisions. The posterior parietal cortex (PPC) emerges as a candidate for processing the rarity information and implementing it in decision-making: (1) previous work in primates has suggested that PPC plays a critical role in processing numerical information [19] and probabilities in uncertain contexts [20–22], and studies in rodents have demonstrated that PPC encodes history information of stimulus, choice, and outcome [23–26], together supporting PPC’s potential role in assessing probabilistic rarity; (2) PPC is also fundamentally involved in decision-making [27–36], and thus has the potential to influence future decisions.

In this study, we isolated the quantitative property of rarity from other factors to investigate its impact on decision-making and learning, using a probabilistic decision-making task with equal magnitude for rare and common outcomes. We found that rare reward strongly influences decision-making in both humans and mice, and the strength of the impact is highly sensitive to how rare the reward is. Optogenetics shows that PPC is required for generating the rarity impact, and *in vivo* physiological recordings demonstrate that rare rewards bias subsequent decisions by enhancing the activity of PPC neurons encoding the biased choice, while suppressing PPC neurons encoding the opposite choice. In addition, learning recruits PPC neurons for stimulus coding, supporting PPC’s essential role in driving stimulus-based optimal decisions. Finally, a data-inspired computational model reproduced the observed behaviors in decision-making and learning under uncertainty.

## RESULTS

### Rare reward has stronger impact on subsequent decisions than common reward in humans

To isolate rarity from other confounding factors, we designed a probabilistic task with identical rewards for common and rare events (Fig. 1A and Fig. S1A). Human subjects press a start (S) button to trigger one of four sounds, then choose either the left (L) or right (R) button to earn monetary rewards. For two sounds (duck/cat), choosing L leads to reward with 0.8 probability and no reward with 0.2 probability; choosing R leads to reward with 0.2 probability and no reward with 0.8 probability. For the other two sounds (canary/frog), contingencies are reversed. Reward is shown as a money symbol upon each choice, otherwise a no-money symbol is shown. We defined common trials as the frequently occurring events (e.g. duck–L–money, or duck–R–no money), and rare trials as the infrequently occurring events (e.g. duck–L–no money, or duck–R–money). Importantly, no single element (stimulus, choice or outcome) is rare *per se*; rarity emerges as the infrequent combination of the three elements. Common and rare trials are pseudo-randomly interleaved, and whether a trial was common or rare is not explicitly indicated (Methods). The optimal strategy is to always select the frequently rewarded choice for each stimulus (i.e. common reward choice) (Fig. S1B). For example, for a duck sound, always choose L. We define these optimal choices as “correct” for assessing task performance. Within a 500-trial limit, 8 of 17 subjects reached expert performance, defined as >85% correct in a 40-trial running window, and an additional four subjects approached this criterion (Fig. 1B).

**Figure 1.**
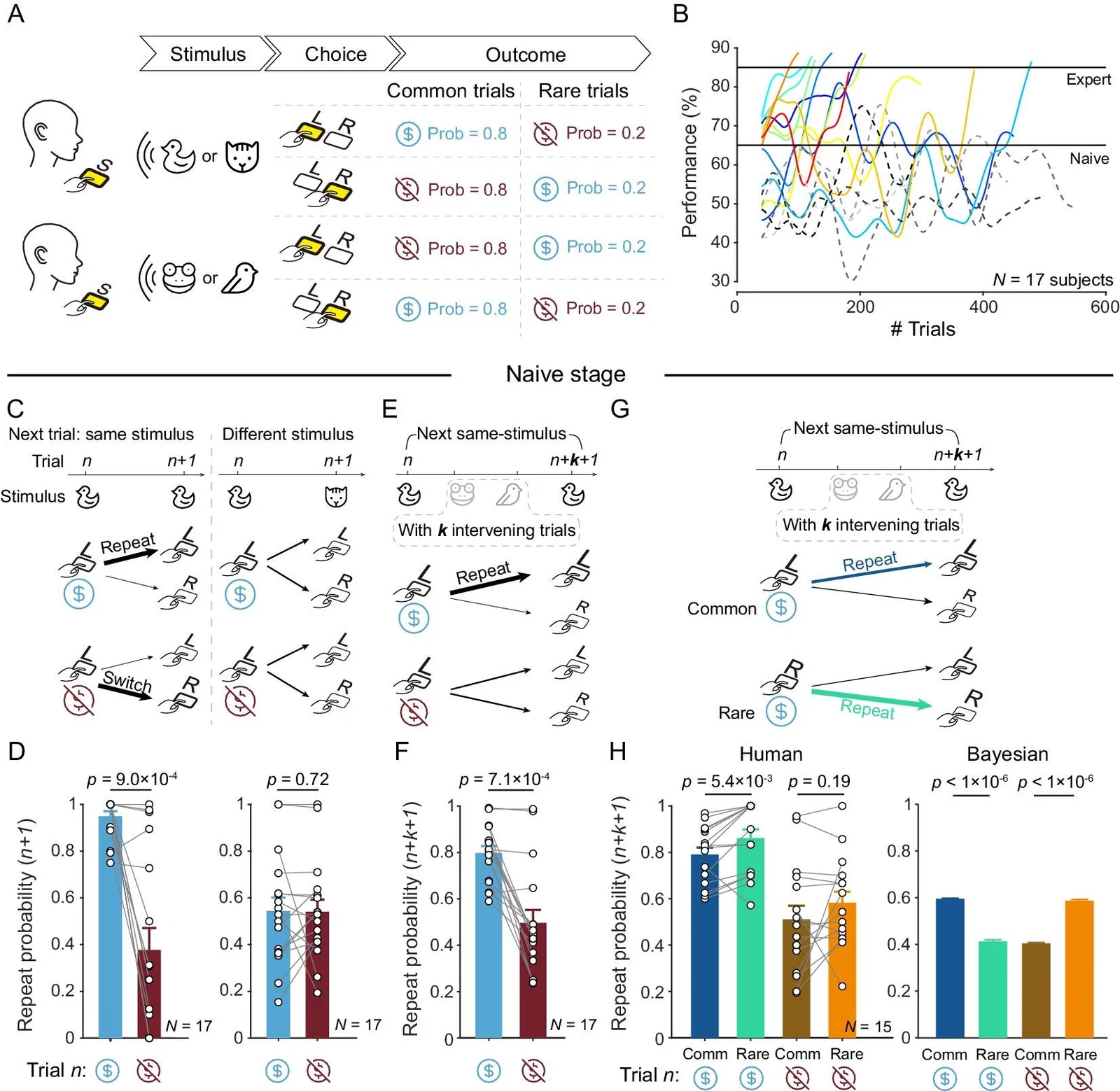
Rare rewards cause a strong decision bias in humans. (A) Schematics showing an auditory probabilistic decision-making task for humans. For duck/cat sound, left choice (L) is associated with 0.8 probability of reward (money symbol), and 0.2 probability of no reward (no money symbol); right choice (R) is associated with 0.2 probability of reward and 0.8 probability of no reward. Probabilities are reversed for canary/frog sound. (B) Performance curve for all subjects, each line representing one subject, calculated as the percentage of correct choices in 40-trial running windows. Naïve, performance <65%. Expert, performance >85%. Dashed lines represent subjects that did not reach expert performance. (C) Schematics illustrating choice repeating or switching in the next trial, with the same (left panel) or different (right panel) stimuli. (D) Naïve stage choice repeat probability for the next trial (nRP*_n+1_*) after money reward or no money in trial *n*, for the same (left panel) or different (right panel) stimuli. Humans follow a stimulus-based win-stay, lose-switch strategy for trial *n+1*. Mean ± S.E.M., Wilcoxon signed-rank test. (E) Schematics illustrating choice repeating after reward, and random choice after no reward, for the next same-stimulus trial, with ***k*** intervening trials of other stimuli in between. (F) Naïve stage choice repeat probability for the next same-stimulus trial (sRP***_n+k+1_***) after reward or no reward trial. Humans follow a stimulus-based win-stay strategy, even with intervening trials. Mean ± S.E.M., Wilcoxon signed-rank test. (G) Schematics illustrating choice repeating after common or rare reward for the next same-stimulus trial (trial *n+k +1*), with ***k*** intervening trials in between. (H) Naïve stage sRP***_n+k+1_*** after common reward (blue), rare reward (green), common no reward (brown), and rare no reward (orange), for humans (left panel), and Bayesian optimal model (1000 simulations, right panel). Data from two subjects were excluded from this analysis due to lack of sufficient trials (see Methods for details). Humans are more likely to repeat rare reward choices than common reward choices, deviating from Bayesian optimal. Mean ± S.E.M., Wilcoxon signed-rank test.

To evaluate the impact of past events on future decisions, we focused on the naïve stage (defined as correct performance <65%), before subjects had firmly established the correct stimulus–choice–reward associations. Prior to the task, subjects were verbally instructed to use sound cues to guide decisions. Their behavior confirmed a stimulus-based, “win-stay, lose-switch” strategy (Fig. 1C): after a rewarded choice, they were much more likely to repeat that choice if they again heard the same sound, than if they heard a different sound; after an unrewarded choice, they were more likely to switch choice for the same sound (**n**ext-trial **R**epeat **P**robability, nRP***_n+1_***, Fig. 1D). Notably, the stimulus-based “win-stay” tendency persisted even with intervening trials of other stimuli in between, as shown by high choice **R**epeat **P**robability on the next **s**ame-stimulus trial (sRP***_n+k+1_, k*** denoting intervening trials, Fig. 1E, F). By contrast, the stimulus-based “lose-switch” tendency diminished with intervening trials (Fig. 1E, F), which is likely due to the low negative valence of unrewarded outcomes, which involved no punishment.

Interestingly, despite being physically identical, rare rewards had an even stronger impact on future decisions than common rewards in the naïve stage (Fig. 1G). Whereas reward in general biases choices by raising repeat probability, rare rewards lead to significantly higher sRP***_n+k+1_*** than common rewards (Fig. 1H and Fig. S1C, D). This is counterintuitive, because: (1) common trials occur more frequently, which should result in stronger reinforcement of common reward choices; (2) repeating rare reward choices objectively yields much lower reward expectation (Fig. S1E), deviating from optimal behavior. To directly test whether this rarity-induced bias leads to suboptimal behavior, we constructed a Bayesian model to simulate optimal behavior in this task [4] (Methods, Fig. S1F). The model predicted the opposite of what we observed: higher sRP***_n+k+1_*** following common rewards than rare rewards (Fig. 1H). Conversely, the model accurately predicted lower sRP***_n+k+1_*** following common unrewarded trials than rare unrewarded trials (Fig. 1H), showing that the rarity-induced bias is absent for unrewarded trials. Together, our results demonstrate that rare rewards have a stronger impact than common rewards on future decisions, causing a systematic deviation from optimality.

### A mouse model recapitulates the rarity impact

To investigate the neural mechanisms underlying the rarity impact observed in humans, we developed a behavioral paradigm in mice, by incorporating probabilistic outcomes to a classical two-alternative forced choice (2AFC) task [37] (Fig. 2A). The mouse initiates a trial by poking into the center port to trigger one of two sounds. For stimulus A (5 kHz pure tone), a left choice is associated with 0.8 probability of water reward (common reward), and 0.2 probability of 3-s timeout punishment (rare punishment); a right choice is associated with 0.2 probability of the same water reward (rare reward), and 0.8 probability of the same punishment (common punishment). Contingencies are reversed for stimulus B (13 kHz tone). All mice trained on this task showed a sigmoidal learning curve across sessions (nine mice, 300–600 trials in each session; Fig. 2B and Fig. S2A) [38], and eventually reached expert performance (defined as >80% correct, calculated as the percentage of correct trials in each session), indicating successful stimulus-based learning.

**Figure 2.**
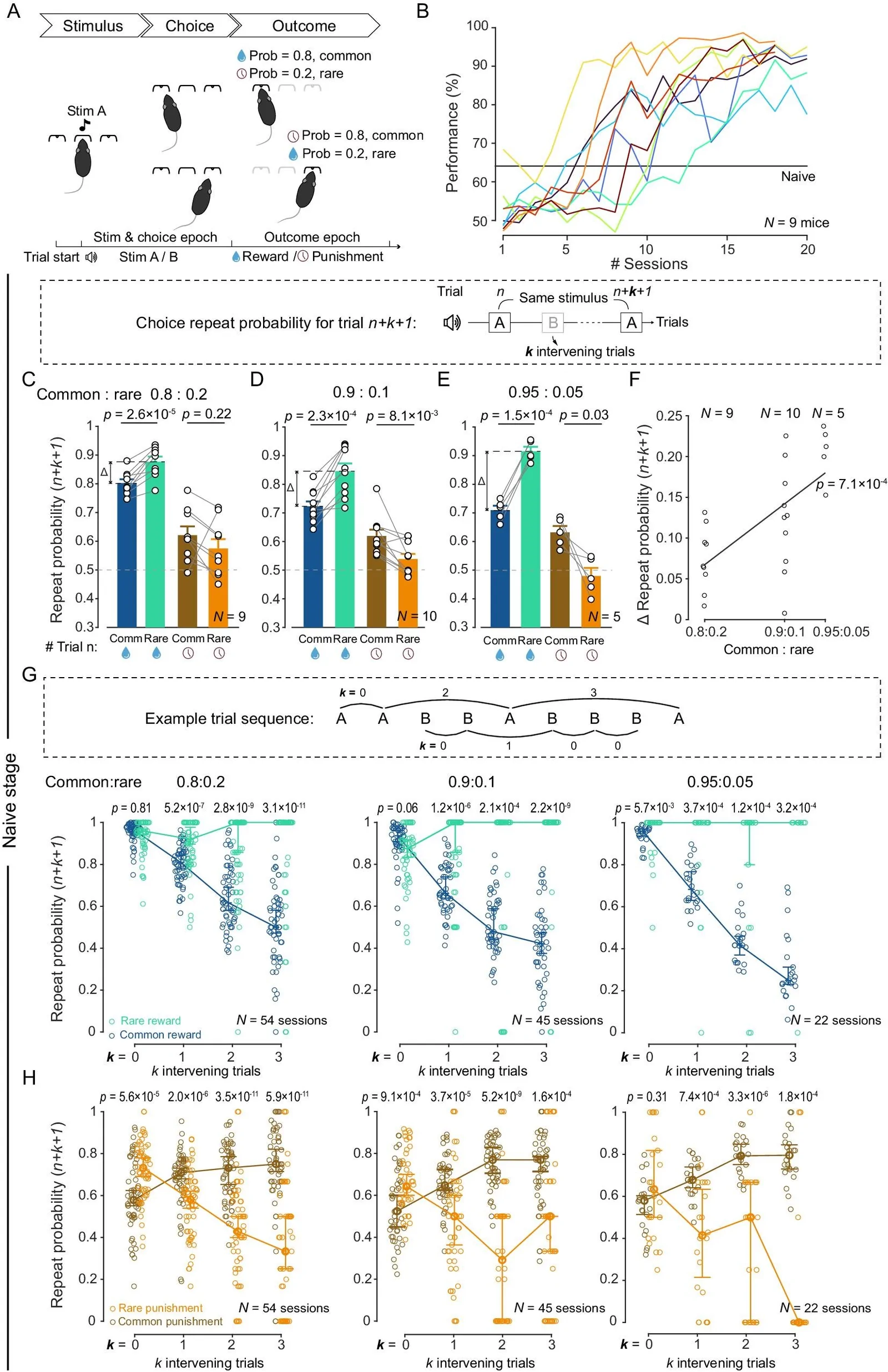
Rare events cause a rare trial ratio-dependent persistent decision bias in mice. (A) Schematics showing mouse auditory probabilistic 2-alternative-forced choice task. For stimulus A, left choice is associated with 0.8 probability of water reward (water symbol), and 0.2 probability of timeout punishment (clock symbol); right choice is associated with 0.2 probability of reward and 0.8 probability of punishment. Probabilities reversed for stimulus B. (B) Performance curve for 9 mice, calculated as the percentage of correct choices in each session. Naïve, performance <65%. (C) Naïve stage choice repeat probability for the next same-stimulus trial (sRP***_n+k+1_***) after common reward, rare reward, common punishment, and rare punishment, at 0.2 rare trial ratio. Mice are more likely to repeat a rare reward choice than a common reward choice, similar to humans. Mean ± S.E.M., paired *t*-test. (D) Naïve stage sRP***_n+k+1_*** at 0.1 rare trial ratio. Mice are more likely to repeat a rare reward choice and switch from a rare punishment choice. Mean ± S.E.M., paired *t*-test. (E) Naïve stage sRP***_n+k+1_*** at 0.05 rare trial ratio. Mice are much more likely to repeat a rare reward choice and switch from a rare punishment choice. Mean ± S.E.M., paired *t*-test. (F) Naïve stage ∆sRP***_n+k+1_*** calculated as the difference in sRP***_n+k+1_*** after rare reward vs. common reward, at 0.2, 0.1 and 0.05 rare trial ratios. Larger positive value indicates stronger bias. One-way ANOVA. (G) Naïve stage sRP***_n+k+1_*** following common or rare reward, as a function of intervening trial number ***k***, at 0.2, 0.1, and 0.05 rare trial ratios. Each data point indicates one session. Note that the influence of common reward (blue) decays over time, while that of rare reward (green) persists or even increases with ***k***. Median ± bootstrapped confidence intervals (CI), Wilcoxon signed-rank test. (H) Naïve stage sRP***_n+k+1_*** following common or rare punishment, as a function of ***k***, at 0.2, 0.1, and 0.05 rare trial ratios. Note that rare punishments (orange) trigger stronger choice switching than common punishments (brown) when intervening trials are present (***k*** ≥ 1). Median ± bootstrapped CI, Wilcoxon signed-rank test.

In the naïve stage (performance <65%, Fig. 2B), rare rewards again led to significantly higher sRP***_n+k+1_*** than common rewards (Fig. 2C and Fig. S2B, C), showing a rarity-induced bias similar to humans. For unrewarded trials, however, as mice experienced timeout punishments that carried negative valence, they were slightly more likely to switch from a rare punishment choice (Fig. 2C), showing a trend opposite to humans, who did not experience punishments.

### Rarity impact is persistent and stronger at lower rare trial ratio

To determine whether the level of rarity influences the strength of the rarity-induced bias, we trained another two groups of mice on tasks with common-to-rare ratios of 0.9 : 0.1 (*N* = 10 mice) and 0.95 : 0.05 (*N* = 5 mice) (Fig. S2A). We found that the rarer the event, the more strongly it influenced future decisions, as measured by sRP***_n+k+1_*** following rare vs. common rewards (Fig. 2C–F and Fig. S2B, C). This graded effect extends to punishments: while rare punishments did not produce a significant bias at 0.2 rare ratio, they led to a highly significant bias at 0.1 and 0.05 ratios, where mice were more likely to switch from a rare punishment than common punishment choice (Fig. 2C–E and Fig. S2D). These results suggest that the rarity impact is a general phenomenon that applies to both positive and negative outcomes, and its strength is sensitive to the rare ratio.

We next investigated the temporal properties of the rarity impact to determine whether it is an immediate effect, which biases only the next trial, or a long-lasting effect that persists over multiple intervening trials. To do this, we examined how sRP***_n+k+1_*** changed with increasing numbers of intervening trials (***k***). When no intervening trials are present (***k*** = 0), both common and rare rewards have strong impact on subsequent same-stimulus trial, shown by high sRP***_n+k+1_*** values (Fig. 2G and Figs S3A and S4A). Notably, only when rare reward is extremely rare (ratio = 0.05), does it induce a stronger impact than common reward (Fig. 2G and Fig. S3A); at other rare ratios (0.2 and 0.1), the rare and common rewards had similar effects at ***k*** = 0 (Fig. 2G and Figs S3A and S4A). This result further confirms that rarity impact is stronger for rarer events. As ***k*** increased, the common reward effect declined rapidly, as expected due to memory decay (Fig. 2G and Fig. S3A). Remarkably, the rare reward effect not only showed no decay, but instead increased to a saturated 100% (Fig. 2G and Fig. S3A), indicating higher persistency than common reward effect. The opposite trends of common and rare reward effects resulted in stronger bias at larger ***k***. A similar temporal pattern was observed for punishments. At ***k*** = 0, common punishment triggered stronger lose-switch than rare punishment, showing optimal behavior (Fig. 2H and Fig. S3B). However, as ***k*** increased, the trend quickly reversed, and rare punishment evoked stronger lose-switch (i.e. lower sRP***_n+k+1_***) than common punishment (Fig. 2H and Fig. S3B). This trend was not observed in humans, possibly due to the absence of punishments in human unrewarded trials (Fig. S4B). These results suggest that rare events leave a stronger memory trace than common events to bias future decisions, when the events carry positive or negative valence.

Furthermore, we found that the level of rarity affected not only the strength, but also the timing of the rarity impact. The higher the common trial ratio, the faster the common reward effect declined with ***k*** (Fig. 2G), indicating faster memory decay as rewards became more common. By contrast, the rarer the reward, the faster sRP***_n+k+1_***increased with ***k*** (Fig. 2G): for the 0.2 rare ratio, median sRP***_n+k+1_*** reached 100% at ***k*** = 2; for 0.1 ratio, it reached 100% at ***k*** = 1; for 0.05 ratio, even with no intervening trial (***k*** = 0), sRP***_n+k+1_*** saturated at 100%. These results demonstrate that while the rarity impact is more apparent with intervening trials, extremely rare events can instantly bias decisions.

Finally, we tested whether this rarity impact could be an artifact caused by stimulus-irrelevant “win-stay” strategy present in naïve mice (i.e. a tendency to repeat a rewarded choice regardless of stimulus; Fig. S5A). According to this strategy, a reward (trial *n*) tends to cause choice repetition in the following intervening trial, which by definition has opposite choice–reward association. If trial n was a rare reward trial, this choice repetition would yield another reward, and in turn cause another choice repetition, whereas if trial *n* was a common reward trial, the choice repetition would yield punishment and cause choice switching. This strategy predicts higher sRP***_n+k+1_*** following rare rewards at ***k*** = 1 (Fig. S5B, gray in Fig. S5C). Same logic extends to larger ***k***. To test this alternative hypothesis, we built a computational model based on this strategy (Methods), and found that its predictions directly contradicted our empirical data (Fig. S5C). First, the model predicts that sRP***_n+k+1_*** for rare rewards should decay with ***k***, while our data showed the opposite (Fig. S5C). Second, the model predicts similar sRP***_n+k+1_*** across rare ratios, contradicting our data for both rare and common rewards (Fig. S5C). Last but not least, the model requires ***k*** ≥ 1 to produce the effect, but we demonstrated that at ***k*** = 0, rare rewards can produce a stronger impact than common rewards when it is sufficiently rare (ratio = 0.05, Fig. 2G). Together, we uncover a robust, long-lasting, and distraction-resilient effect of rare events on future decision-making, whose strength and timing are sensitive to the rarity level.

### PPC is required for generating rarity impact

Previous studies have shown that the PPC encodes probability [20–22] and behavioral history [23–26]. To investigate whether PPC is required in generating the rarity impact, we used optogenetics (eNpHR3.0) to bilaterally inhibit PPC in rare trials, targeting one of two distinct epochs: (1) stimulus&choice epoch (*stim*&*choice-opto_rare_, N* = 5 mice), when mice heard the sound and made the choice (note that the stimulus and choice are temporally inseparable); (2) outcome epoch (*outcome-opto_rare_, N* = 5 mice), when mice received the water reward or timeout punishment (Fig. 3A, B and Fig. S6). A masking yellow light was delivered in all trials to prevent mice from using stimulating light as an additional cue. As rarity manifests only when outcome is delivered, we expected to see an effect by PPC inhibition during the outcome epoch (Fig. 3C). Surprisingly, no effect was observed: *outcome-opto_rare_* mice still showed higher repeat probability (sRP***_n+k+1_***) for rare rewards than common rewards in the naïve stage (Fig. 3D, E and Fig. S7A). Remarkably, PPC inhibition during the stimulus&choice epoch of rare trials (Fig. 3F) completely abolished the rarity impact: *stim*&*choice-opto_rare_* mice showed similar sRP***_n+k+1_*** for rare and common rewards (Fig. 3G–I and Fig. S7A). This result indicates that PPC activity during the stimulus&choice epoch of rare trials is necessary for the rarity impact.

**Figure 3.**
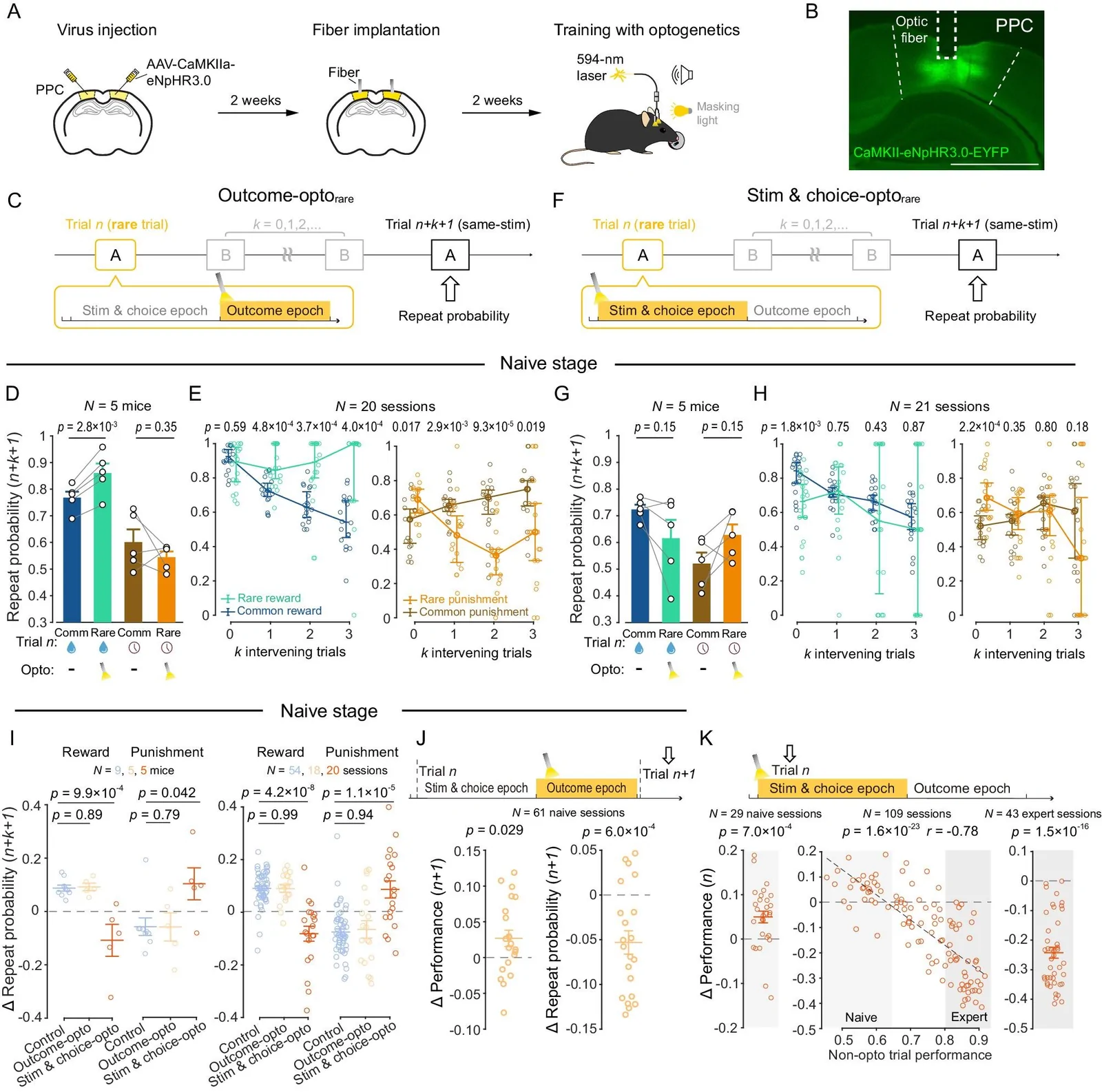
Optogenetic inhibition of PPC during stim&choice epoch eliminates the rarity impact and affects performance. (A) Timeline of optogenetics experiments. Mice were injected with AAV-CaMKII-eNpHR3.0 in bilateral PPC, ∼4 weeks before optogenetic manipulations. (B) Histology image showing the virus expression and the fiber implantation site. (C) Schematic showing optogenetic inhibition during the outcome epoch of rare trials (*outcome-opto_rare_*), focusing on choice repeat probability for the next same-stimulus trial (trial *n+k+1*). (D) Naïve stage sRP***_n+k+1_*** after common reward, rare reward (opto-inhibited), common punishment and rare punishment (opto-inhibited), in *outcome-opto_rare_* mice. Mice are still more likely to repeat rare reward choice than common reward choice, similar to non-optogenetic controls. Mean ± S.E.M., paired *t*-test. (E) Naïve stage sRP***_n+k+1_*** as a function of ***k***, following reward (left panel) and punishment (right panel), in *outcome-opto_rare_* mice. Again, similar to non-optogenetic controls, rare rewards trigger stronger choice repeating and rare punishments trigger stronger switching when intervening trials are present (k ≥ 1). Median ± bootstrapped CI, Wilcoxon signed-rank test. (F) Schematic showing optogenetic inhibition during the stimulus&choice epoch of rare trials (*stim&choice-opto_rare_*), focusing on naïve stage sRP***_n+k+1_***. (G) Naïve stage sRP***_n+k+1_*** in *stim&choice-opto_rare_* mice. Note that these mice no longer show higher sRP***_n+k+1_*** after rare rewards, indicating elimination of the rarity impact. Mean ± S.E.M., paired t-test. (H) Naïve stage sRP***_n+k+1_*** as a function of ***k***, following reward (left) and punishment (right), in *stim&choice-opto_rare_* mice. Rare outcomes have similar effects as common outcomes at k ≥ 1, confirming elimination of rarity impact. Median ± bootstrapped CI, Wilcoxon signed-rank test. (I) Naïve stage ΔsRP***_n+k+1_*** following reward and punishment, in non-opto control, *outcome-opto_rare_* and *stim&choice-opto_rare_* mice. Left panel, each data point represents one mouse. Right panel, each data point represents one session. Note that PPC inhibition during the stim&choice epoch, but not outcome epoch, eliminated the rarity impact. Mean ± S.E.M., unpaired *t*-test. (J) Top, schematic showing *outcome-opto_rare_*, focusing on the performance and the repeat probability of the next trial (trial *n*+1), regardless of stimulus type. Bottom left, the difference between performance (Δperformance) in trial *n* + 1 when trial *n* was inhibited vs. when trial *n* was not inhibited. Positive value indicates improved performance when trial n was inhibited. Bottom right, the difference between nRP*_n+1_* when trial n was inhibited vs. when trial *n* was not inhibited. Negative values indicate reduced repeating when trial n was inhibited. PPC inhibition before stimulus onset improves performance in the naïve stage. Mean ± S.E.M., *t*-test. (K) Top, Schematic showing the optogenetic inhibition during the stim&choice epoch of rare trials (*stim&choice-opto_rare_*) or common trials (*stim&choice-opto_comm_*), focusing on the performance of the inhibited trials. Bottom center, the difference between performance of inhibited trials and that of non-opto trials in the same session (Δperformance), as a function of non-opto trial performance. Shaded areas represent sessions in the naïve and expert stage, respectively. Pearson’s correlation. Bottom left, Δperformance in the naïve stage. Bottom right, Δperformance in the expert stage. Positive values indicate improved performance, and negative values indicate impaired performance, when trial *n* is inhibited. PPC inhibition during stimulus&choice epoch improves performance in the naïve stage and impairs performance in the expert stage. Mean ± S.E.M., *t*-test.

This effect was validated in a separate group of mice (*N* = 5) with alternating optogenetic (*stim*&*choice-opto_rare_*) and control sessions. We observed significantly reduced rarity impact in optogenetic sessions than control sessions in the same mice (Fig. S7B). To further determine if PPC inhibition affects the rarity impact specifically, or the reward-induced choice repetition in general, we inhibited PPC during the stimulus&choice epoch of a subset of common trials (*stim/-opto_comm_, N* = 5). We found that PPC inhibition does not alter common reward influence (Fig. S7C), as shown by similar sRP***_n+k+1_*** in inhibited and non-inhibited trials in the same session. Therefore, PPC is specifically required for the rarity impact, rather than a general reward-induced win-stay tendency. Together, these results indicate that PPC activity during the stimulus&choice epoch, but not the outcome epoch, is required for generating rarity-induced bias.

### PPC neural activity has opposing roles on performance in early vs. late phases of learning

In addition to the rarity-induced bias, our optogenetic experiments also revealed PPC’s contribution to task performance. Although PPC inhibition during the outcome epoch did not affect the rarity impact, we observed significantly improved performance and reduced perseverance (i.e. choice repetition regardless of stimulus or outcome) in the upcoming trials in the naïve stage (Fig. 3J). This aligns with previous work showing PPC inhibition during the inter-trial interval (equivalent to the outcome epoch) reduces history-driven bias [24]. Therefore, disrupting PPC activity during the outcome epoch reduced history-dependent bias that impairs task performance, resulting in improved performance.

Notably, such beneficial effect of PPC inhibition in naïve mice extended into the stimulus&choice epoch [25]: PPC inhibition during this epoch improved performance of the inhibited trials (Fig. 3K). This effect was observed regardless of whether the inhibited trials were common or rare (Fig. S7D), as expected since common and rare trials were indistinguishable before the outcome was delivered. Remarkably, as learning progressed, this effect gradually reversed (Fig. 3K and Fig. S7E). In expert mice, PPC inhibition significantly impaired trial performance (Fig. 3K and Fig. S7F). These results reveal a functional shift in PPC’s role during learning: in naïve mice, PPC encodes history-driven bias which interferes with stimulus-based decision-making; as learning progresses, PPC is increasingly recruited to support stimulus-based decisions.

### Rare reward history modulates PPC activity

To investigate the underlying neural mechanisms, we made extracellular recordings in mouse PPC during task performance (five mice, 103 behavioral sessions, Fig. 4A and Fig. S8A–E), and isolated 3135 single units. We first asked whether PPC neurons encode rarity during the outcome epoch (Fig. 4B). Using three-way analysis of variance (ANOVA; choice × outcome × rarity) on spike rates in a 500 ms time window after outcome delivery, we found that a large fraction of PPC neurons respond differently to left vs. right choices, or rewards vs. punishments, but almost none respond differently to rare vs. common reward or punishment (Fig. 4C and Fig. S9A, B). At the population level, demixed PCA (dPCA) in the same time window also revealed extremely low variance explained by rarity (Fig. 4D and Fig. S9A, B). Thus, PPC neurons encode choices and outcomes, but not rarity. This aligns with the optogenetic result that inhibiting PPC during the outcome epoch does not affect the rarity impact.

**Figure 4.**
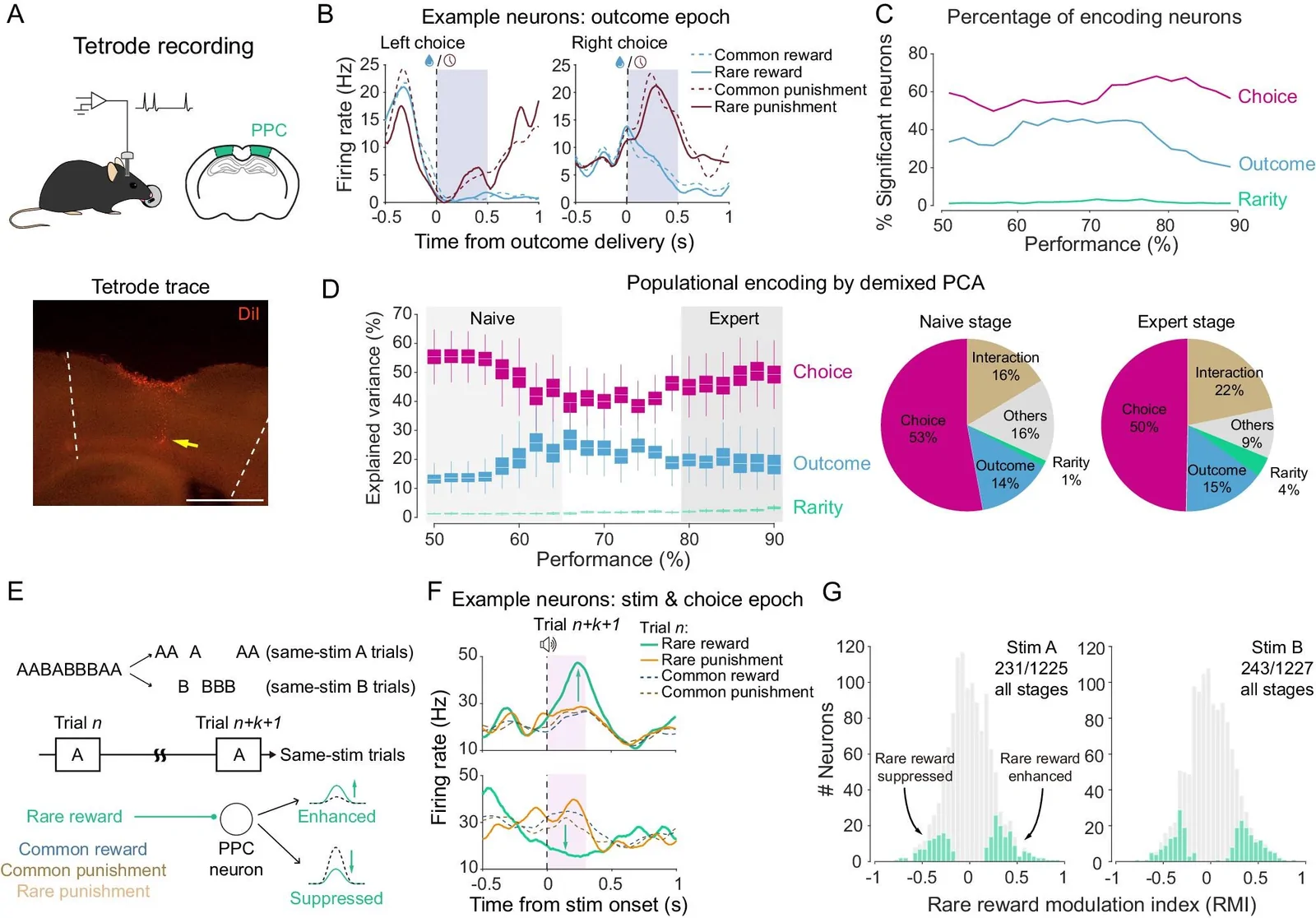
PPC neurons do not encode rarity but are modulated by rare reward trial history. (A) Schematic and histology showing tetrode recording in mouse PPC. (B) Peri-stimulus time histogram (PSTH) of one example neuron, showing different responses to left vs. right choice, reward vs. punishment, but similar response to common vs. rare outcomes. (C) Percentage of significant choice-, outcome-, and rarity-encoding neurons during the outcome epoch, as a function of task performance. Note that many PPC neurons encode choice and outcome, but almost none encode rarity *per se*. (D) Left panel, population variance explained by choice, outcome, and rarity, computed using demixed PCA (dPCA) with 200 neurons (bootstrapped 1000 iterations) recorded in sessions within the performance range of [−5%, +5%]. Box plots show the 25th, 50th (median) and 75th percentiles. Pie charts, explained variance computed using all neurons recorded at <65% (naïve) or >80% (expert) performance. (E) Top, schematic showing separated stimulus A and stimulus B trial sequences. Bottom, schematic showing modulation by preceding rare reward trials on PPC neurons. All ***k*** values are pooled for all the electrophysiology analyses. (F) PSTH of two example neurons in trial *n+k+1*, showing enhanced (top) or suppressed (bottom) activity following rare reward. (G) Histogram showing neuron modulation by preceding stimulus A (left) or stimulus B (right) rare reward, calculated using the receiver operating characteristic (ROC) (bootstrapped 5000 iterations). Colored bars represent significant rarity-modulated neurons.

Then, how does PPC contribute to the rarity impact? As previous studies have shown that PPC neurons encode behavioral history [24,25,39], we investigated whether rare trial history influences PPC activity. As rare reward induced significant behavioral bias in the recorded mice (Fig. S8B, C), we examined how preceding rare reward modulated PPC neurons during the stimulus&choice epoch (critical time window for decision-making) of the next same-stimulus trial. We first separated trials into two trial sequences based on the stimulus type, then further divided trials in each sequence into four groups according to the outcome of the preceding trial (Fig. 4E). Using receiver operating characteristic (ROC) analysis, we computed a rare reward modulation index (RMI) for each neuron, by comparing its spike rates during the stimulus&choice epoch of trials following rare reward, against its firing rates after other trial types (Methods). A positive RMI value indicates enhancement by rare reward history. We observed that ∼20% of PPC neurons were significantly modulated by preceding rare reward, roughly half enhanced and half suppressed (Fig. 4F, G). Together, these results suggest that while PPC does not encode rarity *per se*, its activity during a subsequent decision is modulated by rare trial history.

### Rare trial history bidirectionally modulates choice-encoding PPC neurons to bias choices

Previous studies have shown that PPC can directly guide choices [27,29,33–35]. We thus hypothesize that rare trial history modulates choice-encoding PPC neurons to bias choices (Fig. 5A). To test this, we computed a choice selectivity index (CSI) for each PPC neuron, using ROC analysis on spike rates during the stimulus&choice epoch (Methods). A positive CSI value indicates higher firing rate for right side (“right-selective”) (Fig. S9C–E). Increased activity of right-selective neurons predicts higher right-choice probability, and vice versa for left-selective. A plausible mechanism for the rarity-induced bias is that the rare reward history excites neurons selective for the biased choice, while suppressing neurons selective for the opposite choice, to increase the biased choice probability (Fig. 5A).

**Figure 5.**
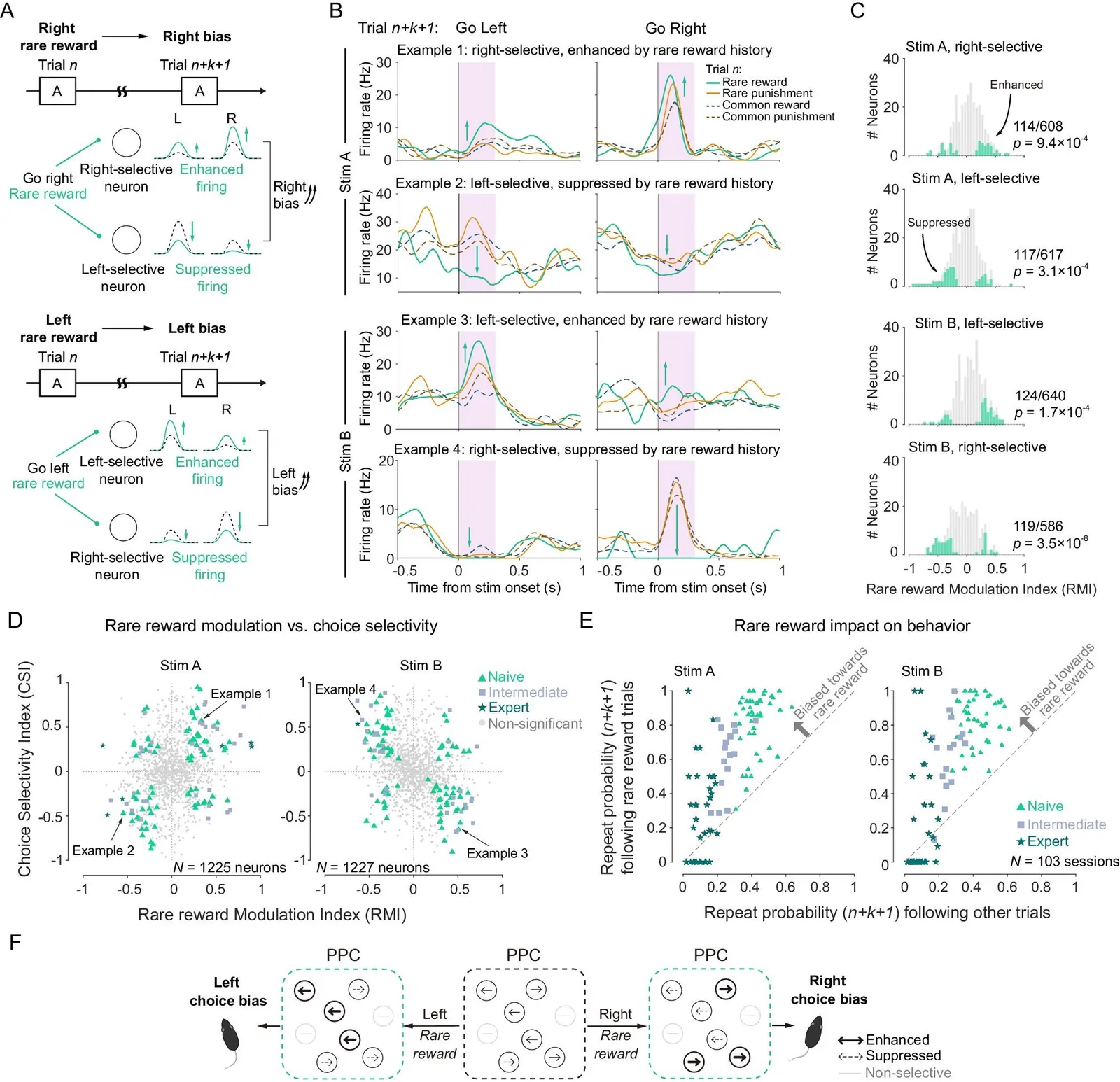
Preceding rare rewards bidirectionally modulate PPC choice-selective neurons to induce choice bias. (A) Top, schematic showing a preceding right rare reward excites a right-selective neuron, and suppresses a left-selective neuron, jointly leading to a right bias. Bottom, schematic showing a preceding left rare reward leads to a left bias. (B) PSTH of 4 example choice-selective neurons, showing modulation by preceding rare rewards, in left and right choice trials. Example 1, a right-selective neuron excited by preceding right rare reward, regardless of the current choice, contributing to increased right-choice probability. Example 2, a left-selective neuron suppressed by preceding right rare reward, regardless of the current choice, contributing to reduced left choice probability. These two example neurons both promote a right bias. Examples 3–4, modulation by preceding left rare rewards contributes to left bias. (C) Choice selectivity index (CSI) histograms showing population results corresponding to the 4 example cells in (B). Wilcoxon signed-rank test. Right rare reward (stim A rare reward) enhances right-selective neurons and suppresses left-selective neurons; left rare reward (stim B rare reward) enhances left-selective neurons and suppresses right-selective neurons. (D) Rare reward modulation index (RMI) plotted against CSI for each neuron recorded in naïve, intermediate, and expert stages. (E) Conditional sRP***_n+k+1_*** following rare reward trials plotted against conditional sRP***_n+k+1_*** following other trials. Before reaching expert stage, choices are strongly influenced by preceding rare reward. (F) Schematic demonstrating how rare rewards bidirectionally modulate choice-encoding neurons to induce choice bias: preceding rare rewards activate neurons encoding the biased choice, and suppress neurons encoding the opposite choice, to promote biased choice.

This is exactly what we found. A rare reward on the right side (i.e. rare reward for stimulus A) enhanced the activity of right-selective neurons during the stimulus&choice epoch of the next stimulus A trial (example 1, Fig. 5B, C), while at the same time suppressed left-selective neurons (example 2, Fig. 5B, C). Such bidirectional modulation jointly leads to a unidirectional right bias. A symmetric effect was observed for stimulus B: a rare reward on the left side enhanced the activity of left-selective neurons and suppressed right-selective neurons in the next stimulus B trial (examples 3 and 4, Fig. 5B, C), leading to a left bias. Importantly, this result was not an artifact of the animal’s biased choices, as we carefully balanced left and right choices in the next trials (trial *n+k+1*) via bootstrapping (Methods). Most rarity-modulated choice-selective neurons were found in the naïve (<65%) and intermediate stages (65%–80%) (Fig. 5D). Consistently, the rarity-induced behavioral bias is reliably observed in these stages, as demonstrated by conditional choice probabilities (Fig. 5E). In the expert stage, the bias is absent in some sessions (data points below the diagonal line, Fig. 5E), indicating that the rarity impact can be overruled by established optimal decision-making. These results provide a neural mechanism for the implementation of the rarity-induced bias: rare trial history biases subsequent decisions by bidirectionally modulating choice-selective PPC neurons (Fig. 5F).

### Learning under uncertainty enhances stimulus encoding of PPC neurons to drive decisions

To understand the neural basis underlying PPC’s opposing roles in decision-making in naïve vs. expert stages, we analyzed how PPC neurons encode different task variables during stimulus&choice epoch across learning. We used three-way ANOVA (stimulus × choice × rare reward history) [27,29,33–35] to identify stimulus, choice, and rare reward history-encoding neurons (Fig. 6A), and used dPCA to quantify population-level variance explained by these factors (Fig. 6B). Both methods consistently showed that as learning progresses, PPC encoding of stimulus increased while rare reward history remained stable.

**Figure 6.**
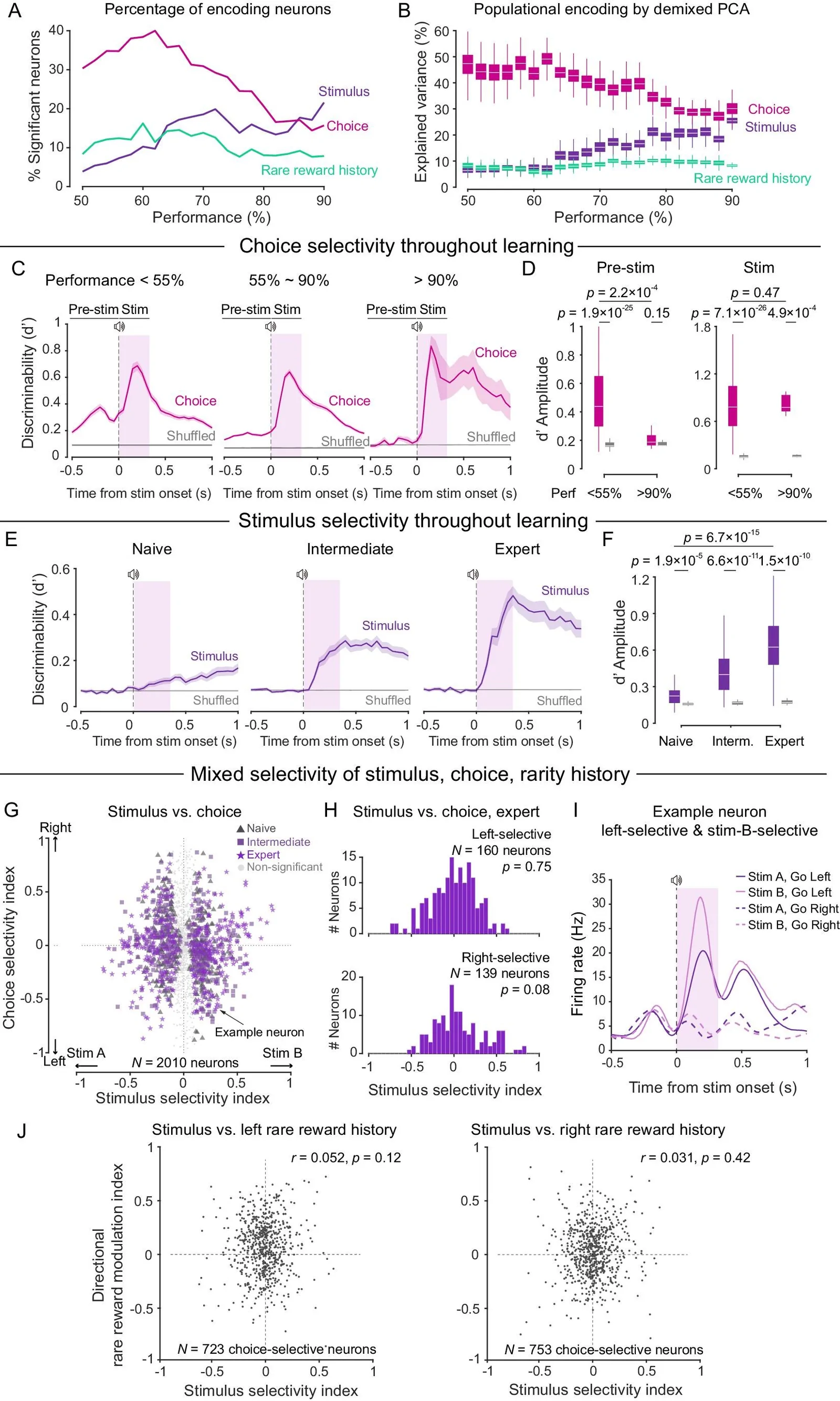
Parallel processing of stimulus and rarity-induced biased choice by PPC neurons. (A) Percentage of significant stimulus-, choice-, and rare reward history-modulated neurons during the stimulus&choice epoch, as a function of task performance. Learning increasingly recruits PPC neurons for stimulus encoding. (B) Population variance explained by stimulus, choice, and rare reward history, computed using demixed PCA with 200 neurons (bootstrapped 1000 iterations) recorded in sessions within the performance range of [−5%, +5%]. Box plots show 25th, 50th (median) and 75th percentiles. (C) Average choice discriminability (d’) for choice-selective neurons, at chance (<55%), 55%∼90%, and high performance (>90%). Shaded areas represent the time window for analysis. Gray lines, shuffled d' (10 000 iterations). (D) Left, amplitude of pre-stimulus choice d' at <55% and >90% performance. Right, amplitude of choice d' during stimulus&choice epoch at <55% and >90% performance. Note that PPC neurons encode intrinsic choice bias when performance is low. Paired *t*-test. (E) Average stimulus d' for stimulus-encoding neurons, at naïve, intermediate, and expert stages. Shaded areas represent the time window for analysis. Gray lines, shuffled d’ (10 000 iterations). (F) Amplitude of stimulus d’ during stim&choice epoch at naïve, intermediate, and expert stages. Note that learning enhances stimulus encoding. Paired t-test and one-way ANOVA. (G) CSI plotted against stimulus selectivity index (SSI) for each neuron recorded in naïve, intermediate, and expert stages. (H) Histogram showing stimulus modulation of choice-selective neurons in the expert stage. Wilcoxon signed-rank test. Note that stimulus A does not preferentially enhance left-selective neurons, nor does stimulus B preferentially enhance right-selective neurons, to promote stimulus-based correct choices. (I) PSTH of one example neuron that is left-selective and enhanced by stimulus B in the expert stage. Note that left choice for stimulus B is incorrect. (J) Directional rare reward modulation index (positive values indicate modulation toward rare reward choice; negative values indicate modulation away from rare reward choice), plotted against stimulus selectivity index, for left rare reward (left panel) and right rare reward (right panel). Pearson’s correlation.

In the naïve stage, especially when performance was around chance level (<55%), PPC activity preceding stimulus onset strongly predicted the animal’s upcoming choice, shown by a high pre-stimulus choice discriminability (d') [40] (Fig. 6C). This result suggests that PPC encodes naïve animal’s intrinsic choice bias. The pre-stimulus choice d' decreases as animal’s performance improves (Fig. 6C, D and Fig. S9F, G), indicating reduced interference with stimulus-based decision-making as animal learns the task. These results are consistent with our finding that PPC inhibition during the outcome epoch (i.e. before upcoming stimulus onset) improves performance in the upcoming trial in naïve animals [41] (Fig. 3J).

In addition to the increasing stimulus-encoding neuron proportion and population variance as learning progresses (Fig. 6A, B and Fig. S9H), stimulus d' of individual neurons during the stimulus&choice epoch also increased (Fig. 6E, F). These results jointly indicate that stimulus-based probabilistic learning recruits PPC neurons for stimulus encoding (Fig. 6A, B, E, F). This provides a neural basis for performance impairment caused by PPC inhibition in the expert stage (Fig. 3K): disrupting PPC activity abolishes the stimulus encoding, which is essential for stimulus-based decision-making in experts.

### Parallel processing of stimulus and rarity-induced choice bias in PPC

Finally, we examined how choice, stimulus, and rare reward history encoding interact in PPC. Note that in the expert stage, mice are highly likely to make left choices for stimulus A and right choice for stimulus B. Following the logic of how rare reward history modulates choice-selective neurons to bias choice, it is possible that stimulus influences choice in the same way. This hypothesis predicts that stimulus A would enhance the activity of left-selective neurons to bias animals toward left choices, while stimulus B would enhance right-selective neurons to promote right choices. As stimulus encoding is enhanced by learning (Fig. 6A, B, E, F), this modulation should be most prominent in the expert stage. We tested this by assessing the stimulus and choice selectivity of all neurons, using the stimulus selectivity index (SSI) and CSI, computed with ROC. A positive SSI value indicates higher firing rate for stimulus B than stimulus A, i.e. enhanced firing in response to stimulus B; conversely, negative SSI indicates enhanced firing for stimulus A (Fig. 6G). If stimulus encoding biases choices toward the correct side (i.e. A–left, B–right), we would expect to see more A-enhanced left-selective and B-enhanced right-selective neurons, than A-enhanced right-selective and B-enhanced left-selective neurons.

Unexpectedly, this was not what we observed (Fig. 6G, H). We found that left-selective neurons were not significantly enhanced by stimulus A, nor were right-selective neurons by stimulus B, in the expert stage (Fig. 6G, H). In other words, even in experts, stimulus does not consistently bias choice toward the correct direction (for an example of a B-enhanced but left-selective neuron, see Fig. 6I). This suggests a mechanism different from the rarity-induced choice bias: although stimulus encoding in PPC contributes to stimulus-based choices, it does not act directly on choice-selective neurons within PPC. Instead, PPC’s stimulus-encoding neurons may be sensory input-defined, and contribute to stimulus-based decisions through downstream motor centers [41–43]. Moreover, we found no correlation between the rarity modulation strength and stimulus selectivity (Fig. 6J), suggesting that stimulus encoding is independent of rarity-induced bias processing. This is further supported by the result that rarity modulation strength is stable throughout learning (Fig. S9I). Together, these results reveal two parallel processes in PPC: rare reward history directly modulates choice-selective neurons within PPC to bias behavior, while stimulus encoding affects choice indirectly, likely through downstream motor circuits.

### A dual-agent model recapitulates rarity-induced decision bias and learning behavior

To examine how the rarity-induced decision bias and stimulus-based decision-making jointly shape learning, we first simulated behavior using four established models: a Bayesian model [4], a win-stay, lose-switch (WSLS) model [44], and two *Q*-Learning models [45]. Although we tested a wide range of parameters, none of the models recapitulated the rarity-induced bias (Fig. S10A–H). We then developed a dual-agent model by combining rarity-based WSLS (r-WSLS) with stimulus-based RL (Methods, Figs S11A and S12A). The first agent implements r-WSLS (Fig. S12B, C) [44], in which WSLS probabilities are updated trial-by-trial based on an estimate of the rarity state [2,46,47]. The second agent implements model-based RL [45], using a multiplicative update rule [38] and different learning rates for reward and punishment [48]. To integrate the two agents, we used normalized decision weights derived from entropy-based metrics in the RL agent [49] (Methods, Figs S11A and S12D). With a fixed parameter set, the model captured the graded rarity impact observed at rare ratios of 0.2, 0.1, and 0.05 (Figs S11B and S12E) and faster learning observed at lower rare ratios (Fig. S11C). Neither agent alone reproduced these results (Fig. S12F, G).

We next asked whether selectively disabling model components would produce behavioral effects resembling optogenetics. Disabling the rarity state in the r-WSLS agent abolished the rarity-induced bias in the naïve stage (Figs S11D and S12H), reproducing the effect of PPC inhibition during the stimulus&choice epoch of rare trials in naïve animals (Fig. 3G–I and Fig. S7A). Disabling the RL agent in the expert stage impaired task performance (Fig. S11E), recapitulating the optogenetic effect in expert animals (Fig. 3K). The model further predicted that reducing the influence of the rarity-sensitive process would accelerate learning. Consistent with this prediction, reanalysis of the optogenetic data showed faster learning in mice receiving PPC inhibition during rare trials (Fig. S11F), supporting its contribution to learning.

## DISCUSSION

To navigate a world where uncertainty is pervasive, one needs to make good decisions in non-deterministic conditions. Using a probabilistic task, we show that in both humans and mice, rare events exert a stronger impact on future decisions than common events, if these rare events carry positive or negative valence. Combining optogenetics, *in vivo* recordings, and computational modeling, we demonstrate that PPC processes multiple functions in parallel: mediating the rarity-induced decision bias, which drives suboptimal choices, and driving stimulus-based decisions, which promotes optimal decisions (Fig. 7).

**Figure 7.**
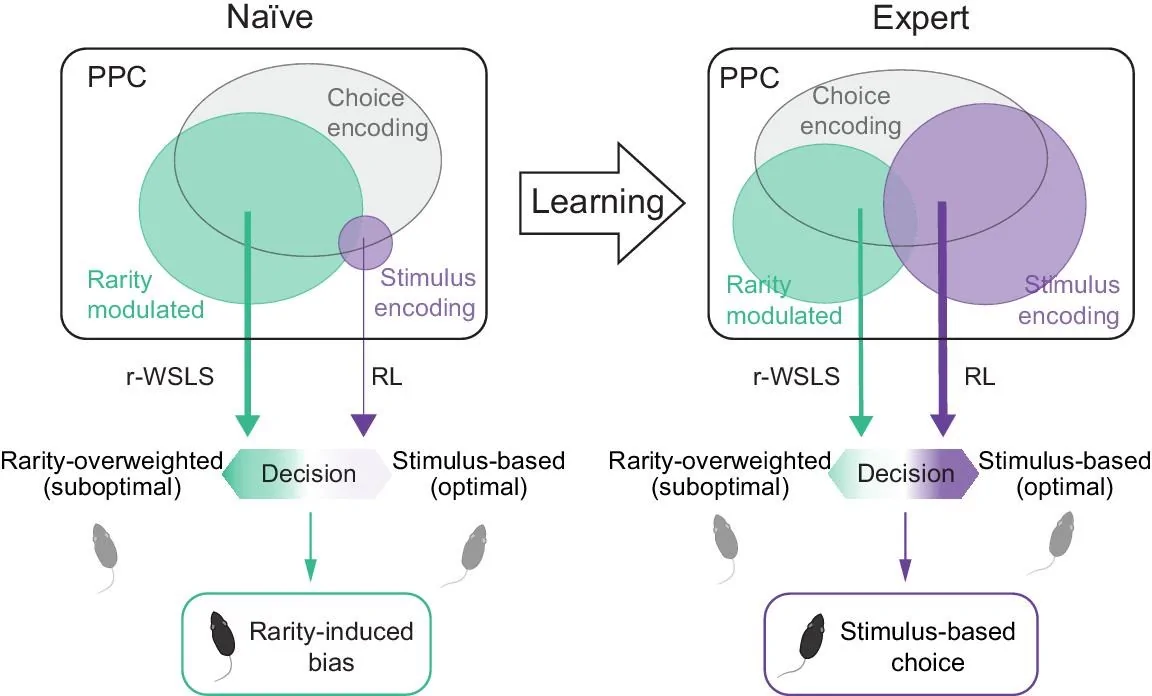
A diagram summarizing PPC’s dual functions in stimulus-based decision-making involving rare probabilities, during the learning process. Circles represent PPC neuronal populations that are rarity-modulated (lower left), stimulus-encoding (lower right), and choice-encoding (upper), at naïve (left panel) and expert (right panel) stages. Animal’s choice for each trial is determined by two decision-making strategies, r-WSLS and RL, which parallel the dual functions of PPC. As learning progresses, the decision weight of r-WSLS decreases while that of RL increases. At the naïve stage, decision-making is strongly influenced by the suboptimal rarity-induced bias, whereas at the expert stage, decision-making is primarily stimulus-based, and animal choices become mostly optimal.

It is well-established that decisions involving rare probabilities are systematically biased, influenced by factors such as recency, saliency, and extremity of rare events [5,9,10,12]. By designing a task that isolated rarity from these factors, we identified a decision heuristic driven by statistical rarity itself. We found that, unlike frequently occurring events, whose influence decayed rapidly with time, rare events left a lasting impact that strengthened over time. Moreover, the rarer the event, the stronger its influence. Importantly, this rarity impact is valence-dependent: if a rare event has minimal value, like the unrewarded trials in the human task, rarity produced no detectable effect. Only when the rare event carries meaningful positive (money for humans, water for mice) or negative (timeout punishment for mice) values did we observe a significant bias. This apparently counterproductive bias may reflect an intrinsic preference for exploration over exploitation in seeking novel valuable resources [8], and elevated vigilance toward unfamiliar threats in uncertain environments.

For well-trained animals performing reinforcement learning tasks, a low-probability outcome deviates more strongly from the expected outcome than a high-probability outcome, hence inducing surprise and generating a larger-magnitude prediction error (PE) [50]. This phenomenon is expected at the expert stage, when stable reward predictions have been established and rare outcomes reliably generate larger PE. However, the rarity-induced bias reported here is most pronounced in the naïve stage, when behavioral performance is low and stable reward predictions have not been established, and thus rare outcomes may not reliably generate larger PE. Moreover, the bias decreases as performance improves, and becomes weaker or undetectable at the expert stage. Finally, the rarity impact persists and even strengthens across intervening trials, suggesting a memory-dependent component. Together, these results suggest that PE magnitude alone may not fully account for the rarity-induced bias. Nevertheless, latent reward predictions may develop before they are expressed at the behavioral level, and in our current task design, the effect of rarity and differences in PE were not fully dissociable. To separate these factors, future studies could implement probabilistic tasks with varying outcome magnitudes across common and rare trials, allowing comparisons to be made under matched outcome probability or PE magnitudes.

PPC has been implicated in integrating sensory evidence to guide perceptual decisions [29,36,51], and encoding trial history information [23–25], but how it contributes to rarity history remains unclear. We found that PPC activity during the stimulus&choice epoch, but not the outcome epoch, of rare trials is necessary for generating the rarity impact. This result suggests that PPC is essential for providing stimulus and choice information to downstream regions for rarity processing, while the outcome information can be provided by other structures such as ventral tegmental area (VTA) or amygdala [50,52]. In addition to generating the rarity impact, PPC also contributes to implementing the bias: rare reward history bidirectionally modulates the activity of choice-selective neurons to bias choices in future trials. Interestingly, although learning enhances PPC’s stimulus encoding, which contributes to optimal decision-making in experts, it does not directly modulate these choice-selective neurons to promote optimal choices. Neurons involved in these two processes are overlapping, and their distinct functions may be determined by connectivity: stimulus-encoding neurons may receive input from the sensory system [25,31,36,53] and influence choice through downstream decision centers [27,36,41–43], whereas history-modulated choice neurons may receive input from regions computing rarity information and directly influence choice. Learning may strengthen connections from stimulus-encoding PPC neurons to decision centers, thereby overriding the influence of history-modulated choice neurons and supporting the behavioral transition from naïve to expert.

Our findings reveal PPC’s multiple roles in decision-making: on one hand, PPC encodes history-related bias and impairs task performance [24,25]; on the other hand, PPC is required for auditory stimulus-based decisions in complex tasks, especially those involving evidence accumulation [31,36]. Future work is needed to identify neural circuits that detect rarity, and reveal how this information is maintained in memory for future decision-making.

## MATERIALS AND METHODS

Human behavioral experiments and animal procedures were approved by the Research Ethics Committee of ShanghaiTech University.

Detailed materials and methods are provided in the Supplementary data.

## Supplementary Material

nwag449_Supplemental_Files
