# Supplementary material for: Posterior parietal cortex mediates rarity-induced decision bias
Source: Natl Sci Rev. 2026 Jul 21;13(18):nwag449. doi: 10.1093/nsr/nwag449 (PMC13637586; doi:10.1093/nsr/nwag449)
Supplement: Supplementary file 1 [file nwag449_supplemental_files.zip › Materials and Methods-updated.pdf]

Materials and Methods

Human subjects

A total of 17 human subjects (6 males and 11 females; mean age ± s.d.: 23.7 ± 5.0, range: 19–40) were recruited in the study. All subjects were healthy and had normal vision and hearing. This study was approved by the Research Ethics Committee of ShanghaiTech University. Every subject signed an informed consent and received monetary compensation for participation. Subjects were informed that the study was related to learning and memory in general, and were instructed to make auditory stimulus-guided decisions for monetary rewards. Subjects were not aware of the probabilistic task design prior to participation.

Human behavioral task

Subjects were seated in front of a computer with a speaker, an LCD monitor, and a custom-made keypad with three keys: "S" for start, "L" for the left choice, and "R" for the right choice. "S" key is controlled by the subject's left hand, and the two choice keys ("L" and "R") are controlled by the subject's right hand.

Before the task, subjects were allowed to adjust the monitor, speaker and keypad to comfortable positions. Auditory stimuli were played from the speaker, controlled using the PsychToolbox software[1]. Key presses were recorded using the Bpod system (Bpod State Machine r2.5, Sanworks) using custom-made software (Matlab). Task instructions were given as texts on the monitor screen. Subjects first went through a pre-task stage. In this stage, auditory stimuli (pure tones, not used in the real task) were played, and subjects were instructed to press the L or R key as specified on the screen upon hearing the auditory stimuli.

Subsequently in the task, subjects were instructed to obtain monetary rewards by pressing the L or R key based on auditory stimuli. Four distinct auditory stimuli are used: duck, cat, canary, and frog sounds (mp3 files downloaded from pixabay.org). The subject presses the "S" key to initiate a trial, triggering the delivery of 1 of 4 auditory stimuli. Upon hearing the stimulus, the subject makes a choice by pressing the "L" or "R" key. As soon as the choice is made, a cartoon showing money earned (rewarded) or money lost (unrewarded) is displayed on the monitor screen. For a duck or cat sound, "L" choice is rewarded at 0.8 probability (common), and "R" choice is rewarded at 0.2 probability (rare); For canary or frog sound, "R" choice is rewarded at 0.8 probability (common), and "L" choice is rewarded at 0.2 probability (rare). Inter-trial interval lasts 2-6 seconds. Common/rare trials were predetermined when the trial sequence was generated. The trial sequence was generated such that stimuli and common/rare trials were pseudorandomly interleaved; rare trials were followed by common trials in approximately 99% of cases, and the same stimulus would not be repeated consecutively for more than 5 trials.

Human behavioral analysis

We defined choices with the higher reward probability as correct choices for calculating task performance: "L" for "duck" and "cat" sounds, and "R" for "canary" and "frog" sounds are considered correct. Performance is calculated as the percentage of correct choices in 20 consecutive trials.

The task was terminated when subjects either reached 85% performance or completed 500 trials, whichever occurred first. To define the naïve stage, we used an empirical performance threshold of 65%. At least 6 *rare reward* trials were included in the analysis for each subject. Two

subjects (1 male and 1 female) encountered only 2 and 3 rare reward trials in the whole session, respectively, and were excluded from the analysis.

The choice Repeat Probability for next same-stimulus trial (RP_{n+k+1}) was defined as the probability of repeating the same choice in the next same-stimulus trial. ΔRP_{n+k+1} for rewarded trials is computed as the difference in RP_{n+k+1} following rare rewarded trials and common reward trials.

To plot ΔRP_{n+k+1} as a function of performance, trials were divided into 40-trial blocks for each subject, and smoothed ΔRP_{n+k+1} was computed by averaging ΔRP_{n+k+1} around performance level with a ±5% window.

To compute RP_{n+k+1} for each intervening trial number k, all trials in the naïve stage were pooled for each subject. Error bars for the median were computed via bootstrapping (5000 iterations).

Animals

Wild-type C57BL/6 mice were purchased from Shanghai Jihui Laboratory Animal Care Co., Ltd. Mice were housed and bred in a 12 h light-dark cycle (7 am - 7 pm light) in the animal facility of ShanghaiTech University. Eight- to 16-week-old male mice were used in the experiments. Animal protocols were approved by the Research Ethics Committee of ShanghaiTech University.

Mouse behavioral task

All behavioral experiments were conducted in a custom-made, electromagnetic shielded, sound-proof booth[2]. A custom-made behavioral chamber has three ports (Mouse port assembly, Sanworks) in the front. Eighteen mice were trained in the task. Mice had limited water access each day starting from three days prior to behavioral training, and were weighed every day to maintain at least 80% of the original body weight. A simple pre-training task was introduced to familiarize the mice with the setup. Specifically, mice were primed with a neutral sound (8 kHz pure tone) to learn the poking sequence: poking into the center port triggers an auditory stimulus, and poking into either side port (left or right) always yields a water reward. This pre-training takes 2 sessions.

In the 2AFC task with probabilistic outcomes, the mouse first pokes into the center port to trigger the delivery of an auditory stimulus (stimulus A: 5 kHz pure tone, 65dB; stimulus B: 13 kHz pure tone, 65dB). The stimulus is preceded by a random (uniform distribution) delay of 50-100ms to prevent the mice from making preliminary decisions, and is terminated when the mouse pokes into a side port (left or right). The outcome (3 μL water reward or 3-second time-out) is delivered immediately as soon as the mouse pokes into a side port. Stimulus A is associated with reward in the left port with 0.8 probability (common) and the right port with 0.2 probability (rare); stimulus B is associated with reward in the right port with 0.8 probability (common) and the left port with 0.2 probability (rare). Inter-trial interval lasts 1-2 seconds. Common/rare trials were predetermined when the trial sequence was generated. Stimuli and common/rare trials were pseudorandomly interleaved; rare trials were followed by common trials in approximately 99% of cases, and the same stimulus would not be repeated consecutively for more than 5 trials.

Mouse behavioral analysis

We defined choices with the higher reward probability as correct choices for calculating task performance: left choice for stimulus A and right choice for stimulus B are considered correct.

Performance was calculated as the percentage of correct trials in each behavioral session. Each session typically contains 300~600 trials. RP_{n+k+1} and ΔRP_{n+k+1} were computed the same way as in humans, except that all calculations were based on sessions, rather than 40-trial blocks. Smoothed ΔRP_{n+k+1} was computed by averaging ΔRP_{n+k+1} around each performance level with a ± 5% window. Empirical RP_{n+k+1} for each intervening trial number k was computed for each individual session. Error bars for the median were computed via bootstrapping (5000 iterations). Theoretical RP_{n+k+1} based on stimulus-irrelevant win-stay lose-switch model was computed for each individual session based on the win-stay/lose-switch probabilities (Pws, Pls) of the session, using the formula below (illustrative examples for $k = 0$ and 1 given in Figure S5B). Pcomm/Prare refers to common/rare trial ratio. Note that a rare trial is always followed by a common trial (additional rule for generating trial sequence, to distribute rare trials more evenly).

k = 0:

RP_{n+k+1}|comm = Pws

RP_{n+k+1}|rare = Pws

k = 1:

RP_{n+k+1}|comm = Pcomm * (Pws * (1 - Pls) + (1 - Pws) * (1 - Pws)) + Prare * (Pws * Pws+(1-Pws) * Pls)

RP_{n+k+1}|rare = Pws*Pws+(1-Pws)*Pls

k = 2:

RP_{n+k+1}|comm = Pcomm * Pcomm * (Pws * (1-Pls) * (1-Pls) + Pws * Pls * (1-Pws) + (1-Pws) * (1-Pws) * (1-Pls)+(1-Pws) * Pws * (1-Pws))+ Pcomm * Prare * (Pws * Pws * (1-Pls)+Pws * (1-Pws) * Pls+(1-Pws) * (1-Pws) * (1-Pls)+(1-Pls) * Pws * Pls) + Pcomm * Prare * (Pws * (1-Pls) * Pws+Pws * Pls * Pls+(1-Pws) * (1-Pws) * Pws + (1-Pws) * Pws * Pls)

RP_{n+k+1}|rare = Pcomm * (Pws * Pws * Pws+Pws * (1-Pws) * Pls+(1-Pws) * Pls * Pws+(1-Pws) * (1-Pls) * Pls) + Prare * (Pws * Pws * (1-Pls)+Pws * (1-Pws) * Pws+(1-Pws) * Pls * (1-Pls) +(1-Pws) * (1-Pls) * (1-Pws))

k = 3:

(1) Following a common reward trial,

a) when the intervening trials are common – common - common:

P_ccc_comm = Pws * (1 - Pls)^3 + Pws * (1 - Pls) * Pls * (1 - Pws) + Pws * Pls * (1 - Pws) * (1 - Pls) + Pws * Pls * Pws * (1 - Pws) + (1 - Pws)^2 * (1 - Pls)^2 + (1 - Pws)^3 * Pls + (1 - Pws)^2 * Pws * (1 - Pls) + (1 - Pws)^2 * Pws^2

b) when the intervening trials are common – rare - common:

P_crc_comm = Pws^2 * (1 - Pls)^2 + Pws * (1 - Pls) * (1 - Pws)^2 + Pws * Pls * Pls * (1 - Pls) + Pws * Pls * (1 - Pws) * (1 - Pls) + (1 - Pws)^2 * Pws * (1 - Pls) + (1 - Pws)^4 + (1 - Pws) * Pws * Pls * (1 - Pls) + (1 - Pws) * Pws * (1 - Pws) * (1 - Pls)

c) when the intervening trials are common – common - rare:

P_ccr_comm = Pws^2 * (1 - Pls)^2 + Pws^2 * Pls * (1 - Pws) + Pws * (1 - Pws) * (1 - Pws) * (1 - Pls) + Pws^2 * (1 - Pws)^2 + (1 - Pws) * Pls * (1 - Pls)^2 + (1 - Pws) * Pls * Pls * (1 - Pws) + (1 - Pws)^2 * (1 - Pls)^2 + (1 - Pws)^2 * (1 - Pls) * Pws

d) when the intervening trials are rare – common - rare:

P_rcr_comm = Pws^3 * (1 - Pls) + Pws^2 * Pls^2 + Pws^2 * (1 - Pws)^2 + Pws^2 * (1 - Pws) * Pls + (1 - Pws) * (1 - Pls) * Pws * Pls + (1 - Pws) * Pls^3 + (1 - Pws)^2 * Pws * (1 - Pls) + (1 - Pws) * (1 - Pls) * Pws * Pls

Combining all 4 conditions:

RP_{n+k+1}|comm = P_ccc_comm * Pcomm * Pcomm + P_ccr_comm * Pcomm * Prare + P_crc_comm * Prare * Prare + P_rcr_comm * Prare * Prare

(2) Following a rare reward trial,

a) when the intervening trials are common – common - common:

P_ccc_rare = Pws^4 + Pws^2 * (1 - Pws) * (1 - Pls) + Pws^2 * (1 - Pws) * Pls + Pws * Pls * (1 - Pws) * (1 - Pls) + (1 - Pws) * Pls * Pws^2 + (1 - Pws)^2 * Pls^2 + (1 - Pws) * (1 - Pls) * Pws * Pls + (1 - Pws) * (1 - Pls)^2 * Pls;

b) when the intervening trials are common – rare - common:

P_crc_rare = Pws^3 * (1 - Pls) + Pws^2 * Pls^2 + Pws^2 * (1 - Pws) * Pls + (1 - Pws)^2 * Pws^2 + (1 - Pws) * Pws * (1 - Pls) * Pls + (1 - Pws) * Pls^3 + (1 - Pws)^2 * (1 - Pls) * Pws + (1 - Pws) * (1 - Pls) * Pws * Pls;
c) when the intervening trials are common – common - rare:
P_ccr_rare = Pws^3 * Pls + Pws^2 * (1 - Pws)^2 + Pws * (1 - Pls) * (1 - Pws)^2 + Pws * Pls * (1 - Pws) * (1 - Pls) + (1 - Pws) * Pws * (1 - Pls) * Pls; + (1 - Pws)^3 * Pls + (1 - Pws)^2 * (1 - Pls)^2 + (1 - Pws) * Pls * (1 - Pls)^2;
Combining all 3 conditions:

RP_{n+k+1}|rare = P_crc_rare * (Pcomm * Prare * (1/(1-Prare * Prare)) + P_ccr_rare * (Pcomm * Prare * (1/(1-Prare * Prare))) + P_ccc_rare * (Pcomm * Pcomm * (1/(1-Prare * Prare)))

Bayesian modeling

To model the decision-making and learning process of humans and mice in the probabilistic task without the rarity impact, we implemented a simple Bayesian reinforcement learning model. This model maintains a belief about the probability of reward associated with choosing the left and right options for a given stimulus, represented by beta distributions. The prior beliefs about the reward probabilities (p_L and p_R) are parameterized by α and β for each choice and stimulus type. On each trial, after the presentation of a stimulus, the model randomly samples from the beta distributions to estimate the expected value for each choice (c_L and c_R). The model then generates a choice by combining these sampled values with added Gaussian noise, such that the left choice is made if $c_L - c_R + \epsilon > 0$, where $\epsilon \sim N(0, \sigma^2)$. After receiving the outcome, the priors p_L and p_R are first multiplied by a discounting factor to represent evidence decay, and then updated based on the Bayesian rule to produce the posterior beliefs, incorporating the observed outcome of the current trial. Briefly, a rewarded choice increases the α parameter, shifting the posterior distribution towards higher probabilities of reward for that choice, while an unrewarded choice increases the β parameter, shifting the distribution towards lower probabilities of reward.

Virus injection and fiber implantation

For the optogenetic inhibition experiments, the 594nm-activated halorhodopsin (eNpHR3.0) fused with yellow fluorescent protein (YFP) is expressed in mouse PPC by stereotaxic injection of adeno-associated virus (AAV9-CaMKII-eNpHR3.0-eYFP, 4.5×10^{13} v.g./mL, OBiO Technology). Mice were anesthetized with isoflurane (1%-1.5%) and positioned in a stereotaxic device (Reward Co.). Body temperature was maintained at 37°C using a small heating pad. Virus was first diluted to 4.5×10^{12} v.g./mL with cell-grade PBS (Solarbio), and injected using a glass pipette with a tip diameter of 20–25 µm via a small skull opening (<0.4 mm^2) with a micro-injector (Nanoject3, Drummond). An aliquot of 300 nL was injected into the PPC of both hemispheres at 1nL/s. The stereotaxic coordinates were: AP -1.94mm, ML ±1.60mm, DV 0.50mm. Optic fibers (0.37 N.A., 200 µm core diameter, 1mm length, Inper) were implanted at a depth of 200 µm below cortical surface at the viral injection sites 2 weeks after virus injection. Following the fiber implantation, mice recover for 1 week before the behavioral training started.

Optogenetic experiments

Twenty mice were used for the optogenetic experiments: N = 5 for PPC inhibition during the stimulus&choice epoch of rare trials (*stim&choice-opto*$_{rare}$), N = 5 for PPC inhibition during the outcome epoch of rare trials (*outcome-opto*$_{rare}$), N = 5 for alternating *stim&choice-opto*$_{rare}$ and non-optogenetic control sessions, N = 5 for PPC inhibition during the stimulus&choice epoch of a subset (10%~20%) of common trials (*stim&choice-opto*$_{comm}$).

Before each session, the implanted optic fiber was connected to a 200μm-core connected fiber optic (Inper) and a rotary joint (Inper) to allow free moving without restriction by the cable. A 589-nm DPSS laser (YL589T6, SLOC) was used, and the laser power was measured before each session to ensure a laser delivery of ~10mW at the fiber tips.

For optogenetic inhibition during the outcome epoch, laser delivery was triggered by the side poke, and lasted until the mouse left the side port or a minimum of 3 seconds in punishment trials. To ensure that mice did not treat the laser illumination as an additional cue, a masking yellow light was delivered during the outcome epoch of all trials.

For optogenetic inhibition during the stimulus&choice epoch: laser delivery was triggered by the center poke, and lasted until the mouse poked into one of the side ports, the same time as the termination of the auditory stimulus. A masking yellow light was delivered during the stimulus&choice epoch of all trials.

In vivo electrophysiology

Custom-made tetrode drives were assembled using polyimide-coated nichrome tetrode wires (PX000004, Sandvik), a 32-channel EIB board (EIB-36-PTB, Neuralynx), and a 3-d printed skeleton that altogether weighed ~0.8 g. Eight tetrodes were gold-plated to impedance <200 kΩ prior to implantation, and implanted in the PPC of both hemispheres. The tetrode tips were dipped in DiI solutions (MB4240-1, meilunbio) to allow post-mortem checking of the implantation site. For tetrode drive implantation, mice were anesthetized with isoflurane (1%-1.5%) and positioned in a stereotaxic device (68803, Reward Co.). Body temperature was maintained at 37°C using a small heating pad. The tetrodes were implanted at the stereotaxic site of AP -1.94mm, ML ±1.60mm, DV 0.50mm. Mice recover in their home cages for 1 week before the training started. Five mice were used for the tetrode recording experiments. Electrophysiological data was collected using the OpenEphys recording system (OEPS-9030, OpenEphys). Before each session, the tetrode drive was connected to a headstage (#C3314, Intan) and the OpenEphys system using a soft connector wire (#C3216, Intan). A custom-made rotator was used to allow free moving of the mice. Before each recording session, each tetrode was independently advanced by 10-40 μm to enable recording of different neurons. To detect spiking activity, the recorded data were bandpass filtered between 600 Hz and 6 kHz, and spikes were thresholded at 40 μV. Clusters were manually sorted using MClust 3.5 (https://redishlab.umn.edu/mclust) by expert experimenters to derive single units. A unit was deemed valid and included in the analysis if it meets all the following criteria[3, 4]: (1) spikes had <1% refractory period violations; (2) cluster separation L-ratio > 0.10; (3) spike presence ratio > 0.95; (4) signal-to-noise ratio > 5.

Electrophysiological data analysis

Single units with mean firing rates of < 1 Hz were excluded from all analyzes. PSTHs were calculated by averaging firing rates in 10-ms bins and smoothed with a Gaussian kernel (σ = 50 ms).

Rare reward modulation index (RMI). To quantify how neural activity of each unit was affected by previous trial outcome (rare reward vs. others), we used an ideal observer decoding based on receiver operating characteristics (ROC) analysis[5]. Using the activity during the stimulus&choice epoch (0~0.3s after stimulus onset), with balanced trial number for left and right choices via bootstrapping, we defined a rare reward modulation index (RMI) as 2 × (auROC – 0.5), which ranged from -1 to 1. RMI>0 indicates higher firing rates following rare reward trials

than following other trials (rare reward enhanced); RMI<0 indicates lower firing rates following rare reward trials than following other trials (rare reward suppressed). A minimum of 3 rare reward trials were required for a unit to be included in the analysis. Significance was assessed via bootstrapping (5000 iterations, $p<0.01$).

Choice selectivity index (CSI). To quantify how each unit's activity encodes the current choice, we estimated auROC on the activity of the stimulus&choice epoch, and defined a choice selectivity index (CSI) as $2 \times (\text{auROC} - 0.5)$. CSI>0 indicates right selectivity, and CSI<0 indicates left selectivity. Significance was assessed via bootstrapping (5000 iterations, $p<0.01$).

Stimulus selectivity index (SSI). To quantify how each unit's activity encodes the current stimulus, we estimated auROC on the activity of the stimulus&choice epoch, and defined a stimulus selectivity index (SSI) as $2 \times (\text{auROC} - 0.5)$. SSI>0 indicates stimulus B selectivity, and SSI<0 indicates stimulus A selectivity. Significance was assessed via bootstrapping (5000 iterations, $p<0.01$).

Directional RMI. For left rare reward, directional RMI = RMI, if CSI < 0 (enhancement of left-selective neurons promotes left choice); directional RMI = -1 × RMI, if CSI > 0 (suppression of right-selective neurons also promotes left choice). For right rare reward, directional RMI = RMI if CSI > 0; directional RMI = -1 × RMI, if CSI < 0.

Three-way ANOVA. (1) To evaluate whether PPC neurons encode rarity during the outcome epoch, we used three-way ANOVA (choice × outcome × rarity) on spike rates in a 0-0.5s time window after outcome delivery, as well as 1.5-2s time window after outcome delivery and -0.5s~0s time window prior to stimulus onset of the upcoming trial. (2) To quantify the significance of encoding stimulus, and choice, and rare reward history, we used three-way ANOVA (stimulus × choice × rare reward history) to fit neural activity of the stimulus&choice epoch (0~0.3s after stimulus onset). A neuron is considered significant if the corresponding $p < 0.01$.

Stimulus and choice discriminability (d'). To compute stimulus d', we computed the mean difference in the smoothed, 50-ms-binned firing rate on the two different trial types (stimulus A vs. B) divided by the square root of their mean variance[6]. Bootstrapping was performed after balancing left and right choice trial numbers. This value was compared with the mean shuffled d' (5000 iterations). To compute choice d', we computed the mean difference in firing rate on the two different trial types (Left vs. Right) divided by the square root of their mean variance, then campared this value with the mean shuffled d' (5000 iterations).

Demixed principal component analysis (dPCA). We followed the procedure in reference[7], by modifying the code provided by the authors (https://github.com/machenslab/dPCA). (1) To evaluate rarity during the outcome epoch, the multi-dimensional input matrices were organized as: neuron ID × outcome type (reward or punishment) × choice type (L or R) × rarity condition (rare or common) × time range × trial ID. The time range used for analysis is 0~0.5s after outcome delivery. (2) To evaluate stimulus, choice, and rare reward history encoding during the stimulus&choice epoch, multi-dimensional input matrices were organized as: neuron ID * stimulus type (A or B) × choice type (L or R) × rarity history (preceding same-stimulus trial is rare reward or not) × time range × trial ID. The time range used for analysis is 0~0.3s after stimulus onset.

A minimum of 3 trials for each combination of variables was required for a neuron to be included in the analysis. For dPCA analysis across performance levels, for each data point at a performance level *perf*, 200 neurons were bootstrapped from a pool of neurons recorded in sessions with performance ∈ [*perf* -5%, *perf* +5%], and repeated 1000 times for the boxplot.

Dual-agent behavioral model

To describe the mouse behavior, we employed a dual-agent approach, combining a rarity-based win-stay-lose-switch (r-WSLS) agent with a stimulus-guided reinforcement learning (RL) agent. Based on the observation that the choice updating WSLS strategy is stimulus-specific, stimulus A trials and stimulus B trials are separately processed in the model[8].

Rarity-based WSLS agent. This agent combines a WSLS-learning model[9] with a rarity state component. The WSLS-learning model allows update in the probabilities of staying and switching. Briefly, the probabilities of repeating a rewarded choice (win-stay) or to switch from an unrewarded choice (lose-switch) change over trials (t), according to:

$$p_{WS}(t+1) = p_{WS}(t) + \theta_{WS}\big(F_{WS} - p_{WS}(t)\big)$$
$$p_{LS}(t+1) = p_{LS}(t) + \theta_{LS}\big(F_{LS} - p_{LS}(t)\big)$$

In the formula above, p_{WS} and p_{LS} represent the probabilities to stay or switch, respectively; θ_{WS} and θ_{LS} represent the update rate of the probabilities; F_{WS} and F_{LS} represent the final, long-term values of the probabilities.

To computationally represent the internal rarity state, we used an unsigned prediction error as the basis for detecting a rare event[10]. We employed a simple value-based approach that is updated using the basic Rescorla-Wagner rule[11],

$$v_{s,a}(t+1) = v_{s,a}(t) + \gamma \cdot \Big(R(t) - v_{s,a}(t)\Big)$$

in which s, a, R represents stimulus, action and outcome, $v_{s,a}(t)$ represents the recency-weighted value of a stimulus-action pair at given trial t, γ represents the update rate. The unsigned discrepancy between actual and predicted value, *i.e.*, the absolute prediction error, is computed as

$$\Delta(t) = \left|R(t) - V_{s,a}(t)\right|$$

We incorporated an internal estimate of rarity, ξ, that represents the agent's belief that the current trial is a common trial or a rare trial. For simplicity, we defined ξ_t to be a binary state value that is a Heaviside step function of the prediction error $\Delta(t)$ at trial t:

$$\xi(t) = H(\Delta(t) - \xi_{thresh})$$

in which $\mathcal{R}_{thresh}$ is the threshold to determine whether a trial is common or rare. We found this estimate sufficient to capture rare events, as it faithfully reflected most of the rare events in the trials.

Based on the behavioral data, we scaled the final probabilities F_{WS} and F_{LS} by the absolute prediction error Δ_t in each trial by the following empirical formula:

$$F_{WS}(t) = p_{perseverance} + \kappa_{WS} \cdot \Delta(\mathrm{t})$$
$$F_{LS}(t) = 1 - \big(p_{perseverance} + \kappa_{LS} \cdot \log(10^2 \cdot \Delta(t) + 1)\big)$$

To simulate the rarity-induced decision bias, we introduced an extra pair of parameters, representing the excessive probabilities of win-stay and lose-switch following rare reward and rare punishment trials. The probabilities are empirically defined as:

$$p_{\Delta WS} = \kappa_{\Delta \mathrm{WS}} \cdot \Delta(t) - \beta_{\Delta WS}$$
$$p_{\Delta LS} = \kappa_{\Delta LS} \cdot \Delta(t)^{\beta_{\Delta LS}}$$

The values of the coefficients $p_{perseverance}$, κ_{WS}, κ_{LS}, $\kappa_{\Delta \mathrm{WS}}$, $\beta_{\Delta WS}$, $\kappa_{\Delta LS}$, $\beta_{\Delta LS}$ are listed in Figure S9a, and are fixed for all simulations regardless of the inputs. To produce a choice in each trial, the r-WSLS agent tracks the last choice and outcome for each stimulus independently, and randomly choose to repeat the last choice (stay) or switch to the other choice (switch), depending on the last rarity state $\xi(t-1)$: if the last trial is a common trial ($\xi(t-1) = 0$), probabilities p_{WS} and p_{LS} are used; if the last trial is a rare trial ($\xi(t-1) = 1$), probabilities $p_{WS} + p_{\Delta WS}$ and $p_{LS} + p_{\Delta LS}$ are used.

Stimulus-guided reinforcement learning (RL) agent. This agent employs a simple Q-Learning model with a multiplicative update rule[12] and distinct learning rates for positive and negative outcomes: if the choice corresponding to the stimulus is rewarded, the association between them (s and a) is strengthened (with learning rate α_{R+}), and the association between the stimulus and the opposite choice (s and a') is weakened (with learning rate α_{R-}); conversely, if the trial was not rewarded, both associations are weakened (with learning rate α_{UR-})[8]. These rules are formulated as:

for rewarded trials,

$$\begin{cases} \delta_{s,a}(\mathrm{t}) = \alpha_{R+} \cdot Q_{s,a}(t) \cdot \left(R(t) - Q_{s,a}(t)\right) \\ \delta_{s,a'}(\mathrm{t}) = \alpha_{R-} \cdot Q_{s,a'}(t) \cdot \left(\epsilon - Q_{s,a'}(t)\right) \end{cases}$$

for unrewarded trials,

$$\delta_{s,*}(\mathrm{t}) = \alpha_{UR-} \cdot Q_{s,*}(t) \cdot \left(\epsilon - Q_{s,*}(t)\right)$$

In these formulae, $\delta_{s,a}(\mathrm{t})$ represents the update for stimulus-action pair (s, a), $Q_{s,a}(t)$ represents the Q-value of the (s, a) pair, and ϵ represents a relatively small noise value ($< 10^{-4}$). Choice from this agent is generated following a noisy epsilon-greedy approach:

$$p(\leftarrow) = N(Q_{s,\leftarrow} - Q_{s,\rightarrow}, \sigma^2)$$

Entropy-based mixture-of-agents model. To integrate the two agents to produce a unified model, we defined normalized decision weights as the probabilities of following each agent's choices. Based on the recording data that: 1) rarity-modulated neurons were constantly present in the PPC, paralleling the persistence of the suboptimal rarity impact throughout learning; while 2) the stimulus-encoding capacity of PPC neurons increases, paralleling the performance improvement as learning progresses, we reasoned that the learning process is mainly driven by the RL agent. Thus, we employed an entropy-based metric[13]. To do so, we first transformed the Q-values into probability distributions using SoftMax function as:

$$P(s, a) = \frac{exp(Q(s, a)/\tau)}{\sum_a exp(Q(s, a)/\tau)}$$

Then we computed the Shannon entropy over the distributions as:

$$H(s) = -\sum P(s, a) \log(P(s, a))$$

Finally, we used the entropy as the decision weight of the RL agent. Fixed decision weight was used for the r-WSLS agent (always 1) before normalization. The final choice is randomly sampled from these choices with the decision weights as the probability distributions.

Statistics

The normality of data was first examined using Lilliefors test. For normally distributed data, paired t-test or t-test were used for paired and unpaired data, respectively. For non-normal data, Wilcoxon signed-rank test or Wilcoxon ranksum test were used for paired and unpaired data, respectively. Statistical tests used in electrophysiological analyzes were described in detail in the "**Electrophysiological data analysis**" section.

Data and code availability

All code is available at https://github.com/YangYangLab. All data and materials are available upon reasonable request.

References

1. Brainard, D.H., *The Psychophysics Toolbox.* Spat Vis, 1997. **10**(4): p. 433–6.
2. Solari, N., et al., *Open Source Tools for Temporally Controlled Rodent Behavior Suitable for Electrophysiology and Optogenetic Manipulations.* Front Syst Neurosci, 2018. **12**: p. 18.
3. Schmitzer-Torbert, N., et al., *Quantitative measures of cluster quality for use in extracellular recordings.* Neuroscience, 2005. **131**(1): p. 1–11.
4. Király, B., et al., *In vivo localization of chronically implanted electrodes and optic fibers in mice.* Nature Communications, 2020. **11**(1): p. 4686.
5. Feierstein, C.E., et al., *Representation of spatial goals in rat orbitofrontal cortex.* Neuron, 2006. **51**(4): p. 495–507.
6. Constantinople, C.M., et al., *Lateral orbitofrontal cortex promotes trial-by-trial learning of risky, but not spatial, biases.* Elife, 2019. **8**.
7. Kobak, D., et al., *Demixed principal component analysis of neural population data.* eLife, 2016. **5**: p. e10989.
8. Fusi, S., et al., *A neural circuit model of flexible sensorimotor mapping: learning and forgetting on multiple timescales.* Neuron, 2007. **54**(2): p. 319–33.
9. Worthy, D.A. and W. Todd Maddox, *A comparison model of reinforcement-learning and win-stay-lose-shift decision-making processes: A tribute to W.K. Estes.* Journal of Mathematical Psychology, 2014. **59**: p. 41–49.
10. Rouhani, N. and Y. Niv, *Signed and unsigned reward prediction errors dynamically enhance learning and memory.* eLife, 2021. **10**: p. e61077.
11. Recorla, R.A., & Wagner, A. R. , *A Theory of Pavlovian Conditioning: Variations in the Effectiveness of Reinforcement and Nonreinforcement. .* 1972.
12. Bathellier, B., et al., *A multiplicative reinforcement learning model capturing learning dynamics and interindividual variability in mice.* Proceedings of the National Academy of Sciences, 2013. **110**(49): p. 19950–19955.
13. Trepka, E., et al., *Entropy-based metrics for predicting choice behavior based on local response to reward.* Nature Communications, 2021. **12**(1): p. 6567.
