# Supplementary material for: Posterior parietal cortex mediates rarity-induced decision bias
Source: Natl Sci Rev. 2026 Jul 21;13(18):nwag449. doi: 10.1093/nsr/nwag449 (PMC13637586; doi:10.1093/nsr/nwag449)
Supplement: Supplementary file 1 [file nwag449_supplemental_files.zip › Supplementary Figures.docx]

**Figure S1**

**Figure S1.** **Example trial sequence, modeling and behavioral results of the human task**

(**A**) An example trial sequence showing the stimuli, subject’s choices, outcomes, and trial types (comm trial or rare trial) in the human probabilistic task. Highlighted trials represent stimulus-based win-stay (choice repeat probability for same-stimulus trials = 1) and stimulus-based lose-switch (choice repeat probability for same-stimulus trials = 0), with *k* intervening trials in between.

(**B**) Decision-making strategies and corresponding overall reward probabilities. To achieve maximal rewards, the optimal strategy is to always choose the common reward side, yielding a maximal reward probability of 80%. Other choice strategies produce lower reward rates. The reward probabilities were calculated by averaging the reward rates of 100 simulations using models based on the corresponding strategies.

(**C**) All stage sRP***_n+k+1_*** following rare and common reward, across block performance. Each data point represents a block.

(**D**) The difference between all stage sRP***_n+k+1_*** following rare and following common reward (∆sRP***_n+k+1_***), as a function of block performance. Positive values indicate choice bias towards rare rewards.

(**E**) The relationship between theoretical reward probability and choice repeating probabilities for the next same-stimulus trial (sRP***_n+k+1_***), for rare (green) and common (blue) reward.

(**F**) Left, schematic showing the Bayesian optimal model. Right, simulated probabilities of making common reward (correct) and rare reward (incorrect) choices, using the Bayesian model.

**Figure S2**

**Figure S2. Mouse behavior results**

(**A**) Learning curves of all mice in 3 different common:rare groups, fitted to sigmoidal curves. Each point represents one session, and each curve represents one mouse.

(**B**) All stage sRP***_n+k+1_*** following rare and common reward, across session performance, for rare trial ratio = 0.2 (left), 0.1 (middle), and 0.05 (right). Each data point represents a session.

(**C**) All stage ∆sRP***_n+k+1_*** as a function of session performance, for rare trial ratio = 0.2 (left), 0.1 (middle), and 0.05 (right). Lines indicate the mean ∆sRP***_n+k+1_*** around each performance level. Each data point represents a session.

(**D)** The difference in naïve stage sRP***_n+k+1_*** after rare punishment vs. common punishment, at 0.2, 0.1 and 0.05 rare trial ratios. More negative values indicate stronger bias after rare punishment. One-way ANOVA.

**Figure S3**

**Figure S3. Choice repeat probability of same-stimulus trials for different intervening trial numbers, for mice**

**(A)** All stage sRP***_n+k+1_*** following rare and common reward, calculated separately for different intervening trial numbers (***k***), as a function of session performance. Each point represents one session. Top row, rare trial ratio = 0.2. Middle row, rare trial ratio = 0.1. Bottom row, rare trial ratio = 0.05. In the naïve stage, sRP***_n+k+1_*** of rare reward trials persists over ***k***, while sRP***_n+k+1_*** of common reward trials decays quickly with ***k*** (more quickly for higher common trial ratio).

**(B)** All stage sRP***_n+k+1_*** following rare and common punishment trials, calculated separately for different **k**, as a function of session performance. Each point represents one session. Top row, rare trial ratio = 0.2. Middle row, rare trial ratio = 0.1. Bottom row, rare trial ratio = 0.05. In the naïve stage, rare punishments trigger stronger switching than common punishments at ***k*** ≥ 1.

**Figure S4**

**Figure S4, Rare rewards cause persistent decision bias in humans**

(**A**) Naïve stage sRP***_n+k+1_*** following rare and common reward, as a function of ***k***, for humans. Each data point represents one subject. Median ± bootstrapped CI. *N* = 15 subjects.

(**B**) Naïve stage sRP***_n+k+1_*** following rare and common unrewarded trials, as a function of k, for humans. Rare unrewarded trials do not induce choice bias. Each data point represents one subject. Median ± bootstrapped CI. *N* = 15 subjects.

**Figure S5**

**Figure S5. Choice repeat probability of same-stimulus trials for different intervening trial numbers, for mice**

(**A**) Naïve stage choice repeat probability for the next trial (nRP*_n+1_*), after reward and punishment trials, at 0.2 (*N* = 9 mice), 0.1(*N* = 10 mice), and 0.05 (*N* = 5 mice) rare trial ratios, regardless of the stimulus of the next trial. Paired t-test.

**(B**) Schematic showing the model based on the stimulus-irrelevant "win-stay" strategy.

(**C**) Top, naïve stage sRP***_n+k+1_*** following rare reward of empirical data (green), and model (grey), as a function of k, at 0.2, 0.1, and 0.05 rare trial ratios. Each data point represents one session. Bottom, naïve stage sRP***_n+k+1_*** following rare punishment of empirical data (blue), and model (grey), as a function of ***k***. Median ± bootstrapped CI.

**Figure S6**

**Figure S6. Histology**

(A) Histology image showing the fiber inhibition site of one example mouse. (B) Implantation sites of all mice in the optogenetic experiments, reconstructed on the mouse brain atlas (Allen Atlas). Scale bar, 1mm.

**Figure S7**

**Figure S7. Optogenetic inhibition of PPC**

(**A**) All stage ΔsRP***_n+k+1_*** as a function of performance for control, *stim&choice-opto_rare_*, and outcome*-opto_rare_* mice. Red and orange data points represent sessions. Red and orange dots on the top indicate significant difference, dot size representing significance level compared to control. Wilcoxon ranksum test.

(**B**) Top, schematic showing experiments with alternating optogenetics (opto) and control (ctrl) sessions for a separate group of mice (*N* = 5). Bottom left, naïve stage sRP***_n+k+1_*** in non-opto (filled bar) and *stim&choice-opto_rare_* sessions (empty bar). Bottom right, naïve stage ΔsRP***_n+k+1_*** in control (cyan) and *stim&choice-opto_rare_* (maroon) conditions. Each data point represents one mouse, wilcoxon signed-rank test. Alternating control and optogenetic sessions confirmed that *stim&choice-opto_rare_* abolished the rarity impact.

(**C**) Top, schematic showing experiments with optogenetic inhibition in a subset (10%-20%) of common trials in a session (*stim&choice-opto_comm_*). Bottom left, naïve stage sRP***_n+k+1_*** in non-opto trials (filled bars) and opto trials (empty bars). Each data point represents one session. Bottom right, naïve stage ΔsRP***_n+k+1_*** in non-opto (cyan) and *stim&choice-opto_comm_* (maroon) sessions. Each data point represents one session. Wilcoxon signed-rank test. Note that PPC inhibition in stimulus&choice epoch of common trials does not affect sRP***_n+k+1_***.

(**D**) The difference between performance of inhibited trials and that of non-opto trials in the same session (Δperformance) in the naïve stage, when inhibited trials were rare trials vs. when inhibited trials were common trials. Positive values indicated improved performance. PPC Inhibition in common and rare trials similarly improved naïve stage performance.

(**E**) Left, Δperformance as a function of non-opto trial performance, when inhibited trials were rare trials. Right, Δperformance as a function of non-opto trial performance, when inhibited trials were common trials.

(**F**) Left, opto trial and non-opto trial performance of an example mouse, which was switched from *outcome-opto_rare_* (after reaching expert performance) to *stim&choice-opto_rare_* protocol. Right, each data point represents one mouse. Wilcoxon signed-rank test. PPC inhibition during the stimulus&choice epoch at expert stage caused an immediate performance impairment, suggesting that this is not a learned artifact.

**Figure S8**

**Figure S8. Behavior results of tetrode recording mice**

(**A**) Learning curves of 5 mice used in tetrode recording experiments across sessions. Each line represents one mouse.

(**B**) Naïve stage sRP***_n+k+1_*** for tetrode recording mice. Each data point represents one mouse. All mice consistently exhibited higher sRP***_n+k+1_*** following rare rewards than common rewards.

(**C**) Naïve stage ∆sRP***_n+k+1_*** for rare vs. common reward of tetrode recording mice vs. control mice.

(**D**) All stage ∆sRP***_n+k+1_*** as a function of performance for tetrode recording mice.

(**E**) Naïve stage sRP***_n+k+1_*** as a function of the number of intervening trials (***k***), for reward (left) and punishment (right), in tetrode recording mice. These mice show similar trend as control mice.

**Figure S9**

**Figure S9. PPC neurons encode choices and outcomes but not rarity**

(**A**) Top, schematic showing the analyzed time period (1.5~2s after outcome delivery). Bottom left, percentage of significant choice-, outcome-, and rarity-encoding neurons, as a function of task performance, determined by three-way ANOVA (p<0.01). Bottom right, population variance explained by choice, outcome, and rarity, calculated using dPCA with 200 neurons (bootstrapped 1000 iterations) recorded during the naïve, learning, and expert stages.

(**B**) Same as (A), but during a different time period: -0.5s~0s prior to the stimulus onset of the upcoming trial.

(**C**) Peri-stimulus time histogram (PSTH) of an example right-selective PPC neuron.

(**D**) Histogram showing choice selectivity index (CSI) of PPC neurons. CSI was calculated using ROC analysis, during the stim&choice epoch (0~0.3s after stimulus onset). Colored bars represent significant choice-selective neurons determined by ROC (bootstrapped 5000 iterations, *p* < 0.01).

(**E**) CSI of individual neurons recorded across performance. Green dots, neurons significantly selective to either left (CSI<0) or right (CSI>0).

(**F**) Average choice discriminability (d’) for choice-encoding neurons, at naïve, learning, and expert stages. Grey areas represent the time window for analyzing pre-stimulus activity. Grey lines, shuffled d’ (10000 iterations).

(**G**) Amplitude of choice d’ 0.3s-0s prior to stimulus onset. Wilcoxon signed rank test for each stage, one-way ANOVA for across stages.

(**H**) Overlap of choice-encoding, stimulus-encoding, and rare reward history*-*modulated neurons at naïve, intermediate, and expert stages. Significance determined by three-way ANOVA (*p* < 0.01).

(**I**) Directional RMI (left and right rare reward conditions combined) as a function of performance.

**Figure S10**

**Figure S10. Simulation results for classic Bayesian, WSLS, and RL models**

(**A**) Simulated naïve stage sRP***_n+k+1_*** for a Bayesian model with slow memory decay. (1000 simulations)

(**B**) Simulated naïve stage sRP***_n+k+1_*** for a Bayesian model with fast memory decay. (1000 simulations)

(**C**) Simulated naïve stage sRP***_n+k+1_*** for a WSLS model with parameters derived from behavioral data. (1000 simulations)

(**D**) Simulated naïve stage sRP***_n+k+1_*** for a WSLS model in which the agent always stays after a win and switches after a loss. (1000 simulations)

(**E**) Simulated naïve stage sRP***_n+k+1_*** for a stimulus-based Q-learning model with low learning rate. (1000 simulations)

(**F**) Simulated naïve stage sRP***_n+k+1_*** for a stimulus-based Q-learning model with high learning rate. (1000 simulations)

(**G**) Simulated naïve stage sRP***_n+k+1_*** for a stimulus-based Q-learning model with higher learning rate for rewarded trials than for punished trials (1000 simulations). Note that the sRP***_n+k+1_*** following rare rewards is not significantly higher than sRP***_n+k+1_*** following common rewards.

(**H**) Simulated naïve stage sRP***_n+k+1_*** for a stimulus-based Q-learning model with a higher learning rate for punishment than reward. (1000 simulations)

**Figure S11**

**Figure S11. A data-inspired dual-agent model recapitulates the decision bias and learning process**

(**A**) Schematic showing the dual-agent model. The model consists of a rarity-based win-stay lose-switch (r-WSLS) agent and a reinforcement learning (RL) agent. In each trial, the r-WSLS agent produces a choice based on the repeat probabilities generated by WSLS model, and modulated by the rarity state (ξ) through a gating mechanism. The rarity state is a binary variable that determines if the previous prediction error is large enough (above a threshold) to generate a rarity-induced bias. The RL agent employs a Q-Learning model that uses SoftMax to produce a choice in each trial. The distribution of the Q-values is converted to the decision weight of the RL agent through entropy-based methods. The final choice is generated using weighted randomization. The outcome is used to update rarity state and the internal short-term value (*v*) in the r-WSLS agent, and the Q-values in the RL agent with learning rate α.

(**B**) Simulated naïve stage sRP***_n+k+1_*** (20 iterations) for 0.2, 0.1, and 0.05 rare trial ratios, recapitulating the rarity impact.

(**C**) Trial number to reach expert stage for 3 rare trial ratios, for model simulation (left) and empirical data (right), showing recapitulation of successful learning.

**(D)** Disabling rarity state in the WSLS agent eliminated the rarity impact in the naïve stage, as shown by sRP***_n+k+1_*** (left) and ∆sRP***_n+k+1_*** (right).

(**E**) Disabling RL agent impairs performance in the expert stage.

(**F**) Trial numbers to reach expert stage (rare trial ratio = 0.2). Right, empirical data showing PPC inhibition during stimulus&choice epoch of rare trials facilitates learning (maroon) compared to control mice (grey). Left, model simulation showing disabling both agents during rare trials facilitates learning. Mean ± s.e.m..

**Figure S12**

**Figure S12. Dual-agent model parameters and modeling results**

(**A**) Parameter values (fixed for all simulations) used in the model.

(**B**) Example trials showing the actual (blue) and model-estimated (red) occurrence of rare trials, with a given trial sequence.

(**C**) Correlation of actual and estimated rare trial occurrences (20 simulations).

(D) Normalized decision weights of the RL agent and r-WSLS agent as a function of trial number. The decision weight of the RL agent increases with learning, while the decision weight of r-WSLS agent is set as fixed before normalization, and decreases with learning after normalization.

(**E**) Simulated all stage ∆sRP***_n+k+1_*** for 0.2, 0.1, and 0.05 rare trial ratios.

(**F**) Left, simulated learning curve; right, simulated naïve stage sRP***_n+k+1_*** for 0.2 rare trial ratio, using the r-WSLS agent alone. This single-agent r-WSLS model can capture the rarity-induced bias but fails to produce successful learning.

(**G**) Left, simulated learning curve; right, simulated naïve stage sRP***_n+k+1_*** for 0.2 rare trial ratio, using the RL agent alone. This single-agent RL model can produce successful learning but fails to replicate the rarity-induced bias in the naïve stage.

(**H**) Simulated all stage ∆sRP***_n+k+1_*** for 0.2 rare trial ratio in *in-silico* inactivation.
